# Supplementary material for: Disentangling molecular mechanisms regulating sensitization of interferon alpha signal transduction
Source: Mol Syst Biol. 2020 Jul 21;16(7):e8955. doi: 10.15252/msb.20198955 (PMC7373899; doi:10.15252/msb.20198955)
Supplement: Supplementary file 1 — Appendix [file MSB-16-e8955-s001.pdf]

Appendix:
Disentangling molecular mechanisms regulating sensitization of interferon alpha signal transduction

Frédérique Kok[1,7#], Marcus Rosenblatt[2#], Melissa Teusel[1,7#], Tamar Nizharadze[1,7], Vladimir Gonçalves Magalhães[3], Christopher Dächert[3,7], Tim Maiwald[2], Artyom Vlasov[1,7], Marvin Wäsch[1], Silvana Tyufekchieva[4], Katrin Hoffmann[4], Georg Damm[5], Daniel Seehofer[5], Tobias Boettler[6], Marco Binder[3], Jens Timmer[2,8,9*°], Marcel Schilling[1*°°], Ursula Klingmüller[1*°°°]

[#] These authors contributed equally

* Shared last authors

Affiliations:

[1] Division Systems Biology of Signal Transduction, German Cancer Research Center (DKFZ), 69120 Heidelberg, Germany

[2] Institute of Physics, University of Freiburg, 79104 Freiburg, Germany

[3] Research Group "Dynamics of early viral infection and the innate antiviral response", Division Virus-associated carcinogenesis, German Cancer Research Center (DKFZ), 69120 Heidelberg, Germany

[4] Department of General, Visceral and Transplantation Surgery, Ruprecht Karls University Heidelberg, 69120 Heidelberg, Germany

[5] Department of Hepatobiliary Surgery and Visceral Transplantation, University of Leipzig, 04103 Leipzig, Germany

[6] Department of Medicine II, University Hospital Freiburg - Faculty of Medicine, University of Freiburg, 79106 Freiburg, Germany

[7] Faculty of Biosciences, Heidelberg University, 69120 Heidelberg, Germany

[8] Signalling Research Centres BIOSS and CIBSS, University of Freiburg, 79104 Freiburg, Germany

[9] Center for Biological Systems Analysis (ZBSA), University of Freiburg, 79104 Freiburg, Germany

Please direct all correspondence to:

°°° Prof. Dr. Ursula Klingmüller, Division Systems Biology of Signal Transduction, German Cancer Research Center (DKFZ), INF 280, 69120 Heidelberg, Germany. Phone: +49 6221 42 4481, Fax: +49 6221 42 4488, e-mail: u.klingmueller@dkfz.de

or:

°° Dr. Marcel Schilling, Division Systems Biology of Signal Transduction, German Cancer Research Center (DKFZ), INF 280, 69120 Heidelberg, Germany. Phone: +49 6221 42 4485, Fax: +49 6221 42 4488, e-mail: m.schilling@dkfz.de

or:

° Prof. Dr. Jens Timmer, Institute of Physics, University of Freiburg, Hermann - Herder Str. 3, 79104 Freiburg, Germany. Phone: +49 761 203 5829, Fax: +49 761 203 8541, e-mail: jeti@fdm.uni-freiburg.de

Contents

Figures 3

Appendix Figure S1 . . . 3
Appendix Figure S2 . . . 4
Appendix Figure S3 . . . 5
Appendix Figure S4 . . . 6
Appendix Figure S5 . . . 7
Appendix Figure S6 . . . 8
Appendix Figure S7 . . . 9
Appendix Figure S8 . . . 11
Appendix Figure S9 . . . 13
Appendix Figure S10 . . . 14
Appendix Figure S11 . . . 15
Appendix Figure S12 . . . 16
Appendix Figure S13 . . . 20

Tables 21

Appendix Table S1 . . . 21
Appendix Table S2 . . . 25
Appendix Table S3 . . . 26
Appendix Table S4 . . . 28
Appendix Table S5 . . . 32
Appendix Table S6 . . . 32
Appendix Table S7 . . . 33
Appendix Table S8 . . . 34

References 36

Figures

Appendix Figure S1

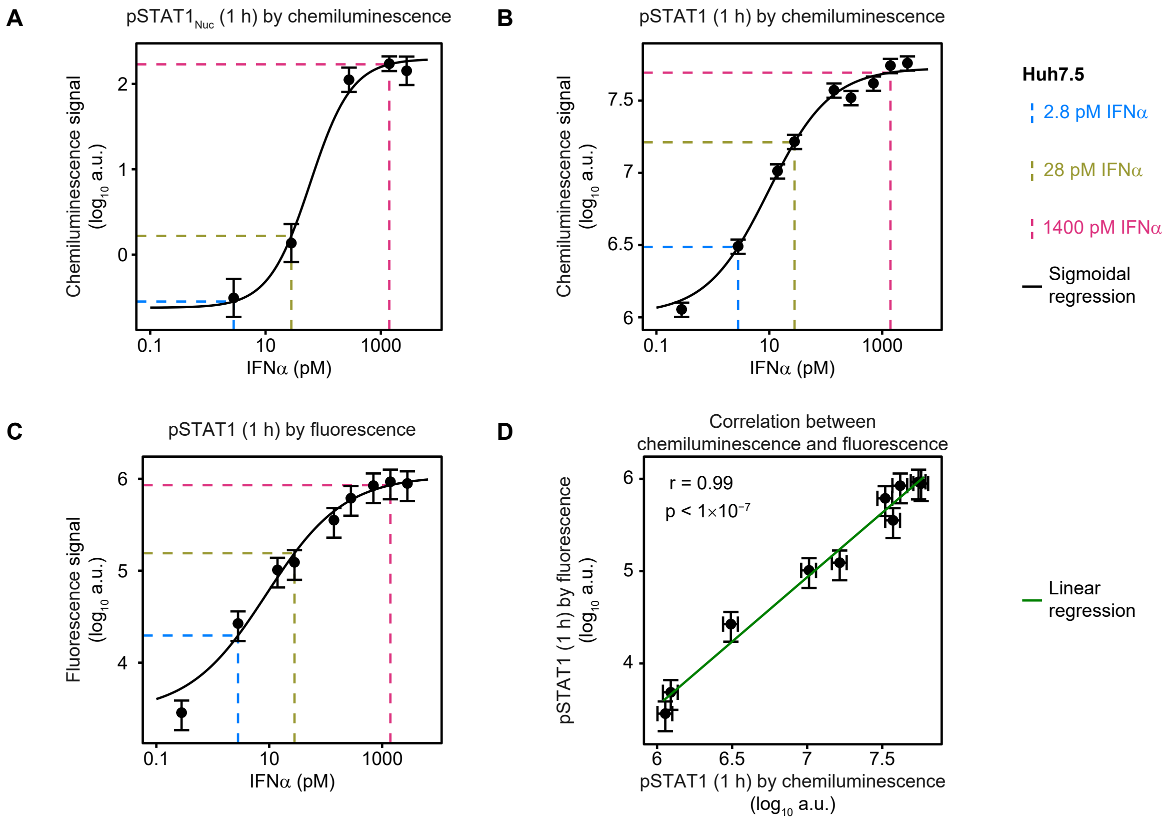

Appendix Figure S1: IFNα dose-dependency of STAT1 phosphorylation and comparison between chemiluminescence and fluorescence
IFNα dose-dependency of STAT1 phosphorylation in Huh7.5 cells measured by chemiluminescence and fluoresence. Cells were seeded 24 hours prior to the start of the experiment. Three hours before stimulation, cells were growth factor-depleted and were subsequently stimulated with the indicated concentrations of IFNα. Nuclear (nuc) protein lysates (A) or total protein lysates (B-D) were collected one hour after the stimulation and phosphorylation of STAT1 was detected by immunoblot utilizing antibodies recognizing STAT1 phosphorylated on tyrosine residue 701. Immunoblot detection was performed with chemiluminescence (A,B) employing a CCD camera device (ImageQuant) or with fluorescence (C) employing a near-infrared fluorescence scanner (Odyssey). Data is approximated with a sigmoidal function and signals corresponding to a low dose (2.8 pM IFNα), a medium dose (28 pM IFNα) and a high dose (1400 pM IFNα) are displayed with dashed lines. pSTAT1 values by fluorescence are correlated to pSTAT1 values by chemiluminescence (D). Pearson correlation coefficient (r) and p-value (p) are indicated.

Appendix Figure S2

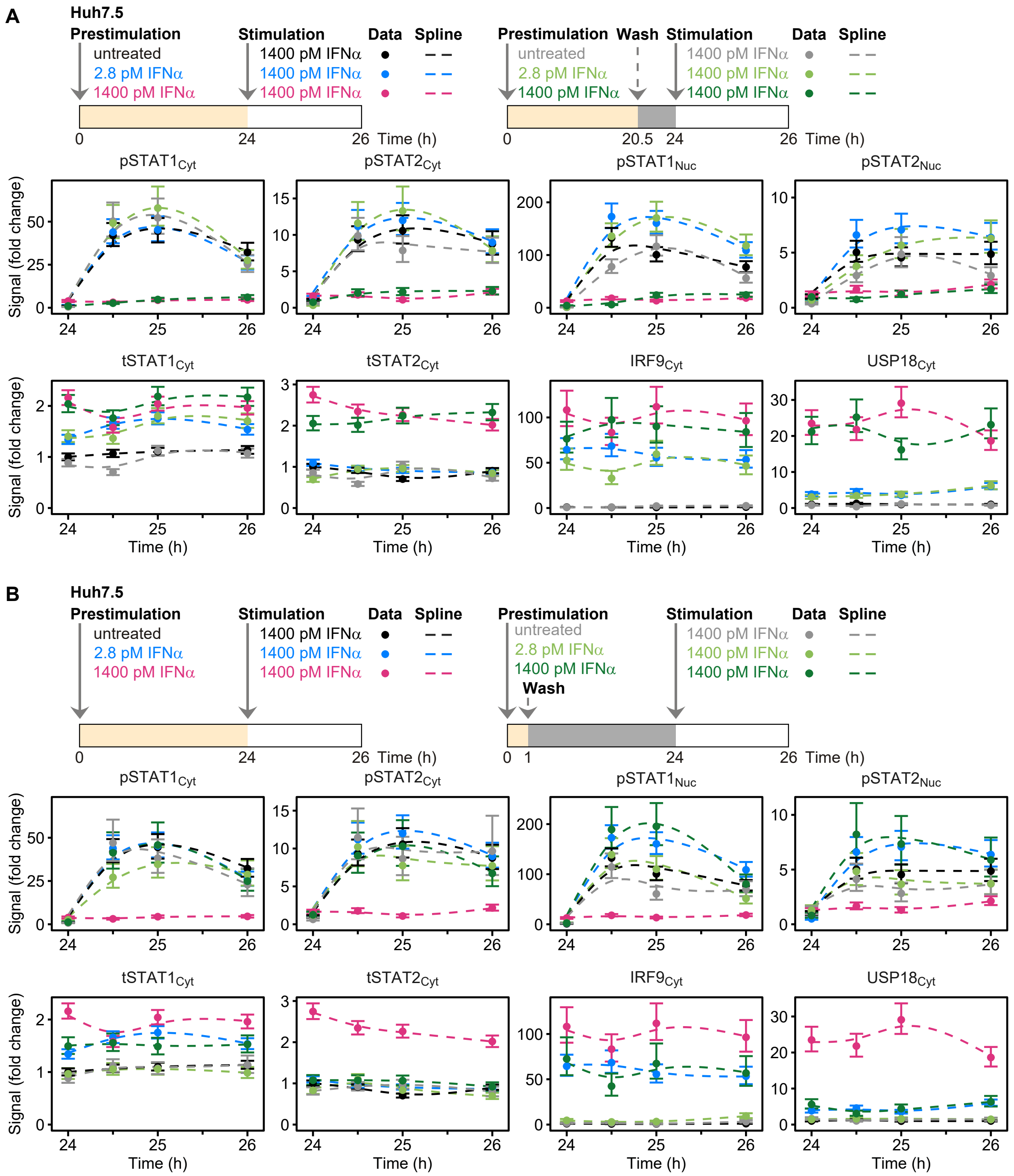

Appendix Figure S2: Dose-dependent IFNα-induced sensitization is independent of ligand presence, but requires ligand exposure over one hour to establish

Huh7.5 cells were growth factor-depleted for three hours and received prestimulation with 2.8 pM IFNα, 1400 pM IFNα or no pretreatment. Cells were washed at 20.5 hours after prestimulation (A) or were washed at one hour after prestimulation (B) before cells were stimulated with 1400 pM IFNα 24 hours after prestimulation. Cytoplasmic and nuclear lysates were subjected to quantitative immunoblotting. IFNα-induced phosphorylation of STAT1 and STAT2 and induction of feedback proteins was detected with chemiluminescence utilizing a CCD camera device (ImageQuant). Data is represented by filled circles with 1σ confidence intervals estimated from biological replicates (N=2 to N=3) using a combined scaling and error model. Dashed lines represent smoothing splines.

Appendix Figure S3

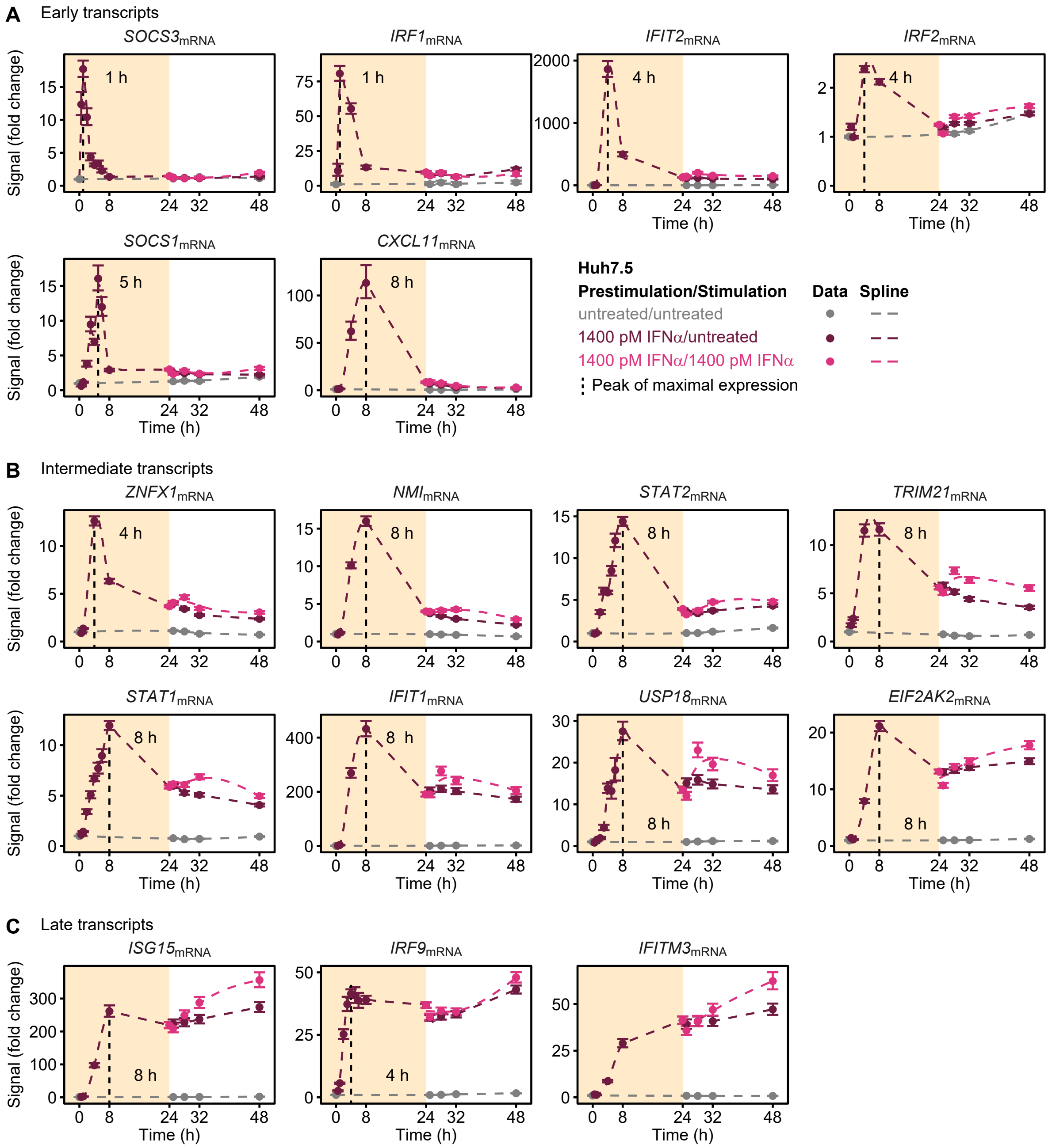

Appendix Figure S3: Dynamics of interferon-stimulated transcripts upon prestimulation with a high IFNα dose

Induction of interferon-stimulated genes upon prestimulation with 1400 pM IFNα (yellow background) and stimulation at 24 hours with 1400 pM IFNα (white background) in Huh7.5 cells, assessed by qRTPCR is shown. RNA levels were normalized to the geometric mean of reference genes GAPDH, HPRT and TBP and are displayed as fold change. Peak of gene expression is indicated. Data points displayed as dots with 1σ confidence interval estimated from biological replicates (N=4 to N=14) using a combined scaling and error model, dashed lines indicate spline.

Appendix Figure S4

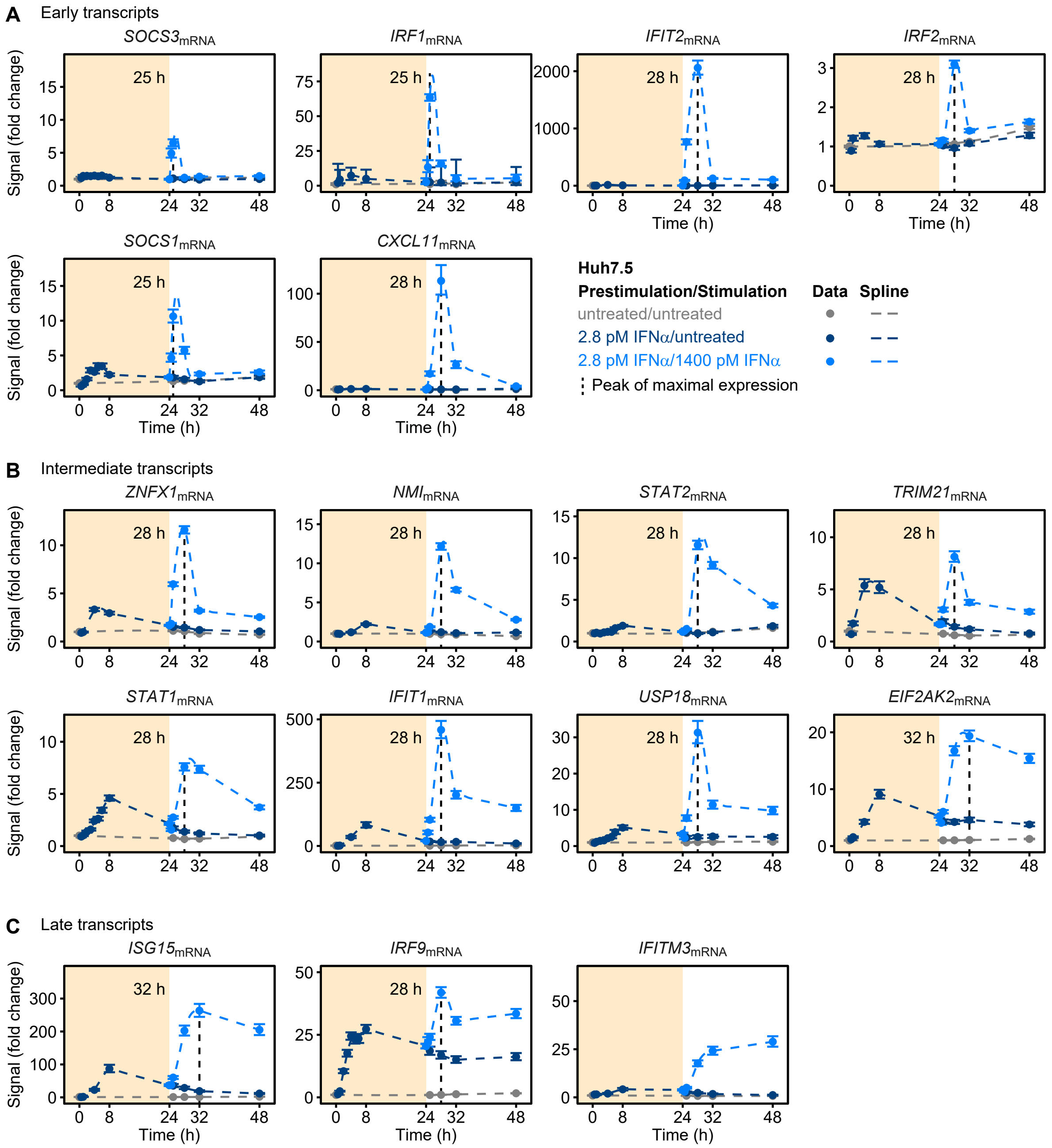

Appendix Figure S4: Dynamics of interferon-stimulated transcripts upon prestimulation with a low IFNα dose

Induction of interferon-stimulated genes upon prestimulation with 2.8 pM IFNα (yellow background) and stimulation at 24 hours with 1400 pM IFNα (white background) in Huh7.5 cells, assessed by qRTPCR is shown. RNA levels were normalized to the geometric mean of reference genes GAPDH, HPRT and TBP and are displayed as fold change. Peak of gene expression is indicated. Data points displayed as dots with 1σ confidence interval estimated from biological replicates (N=4 to N=6) using a combined scaling and error model, dashed lines indicate smoothing splines.

Appendix Figure S5

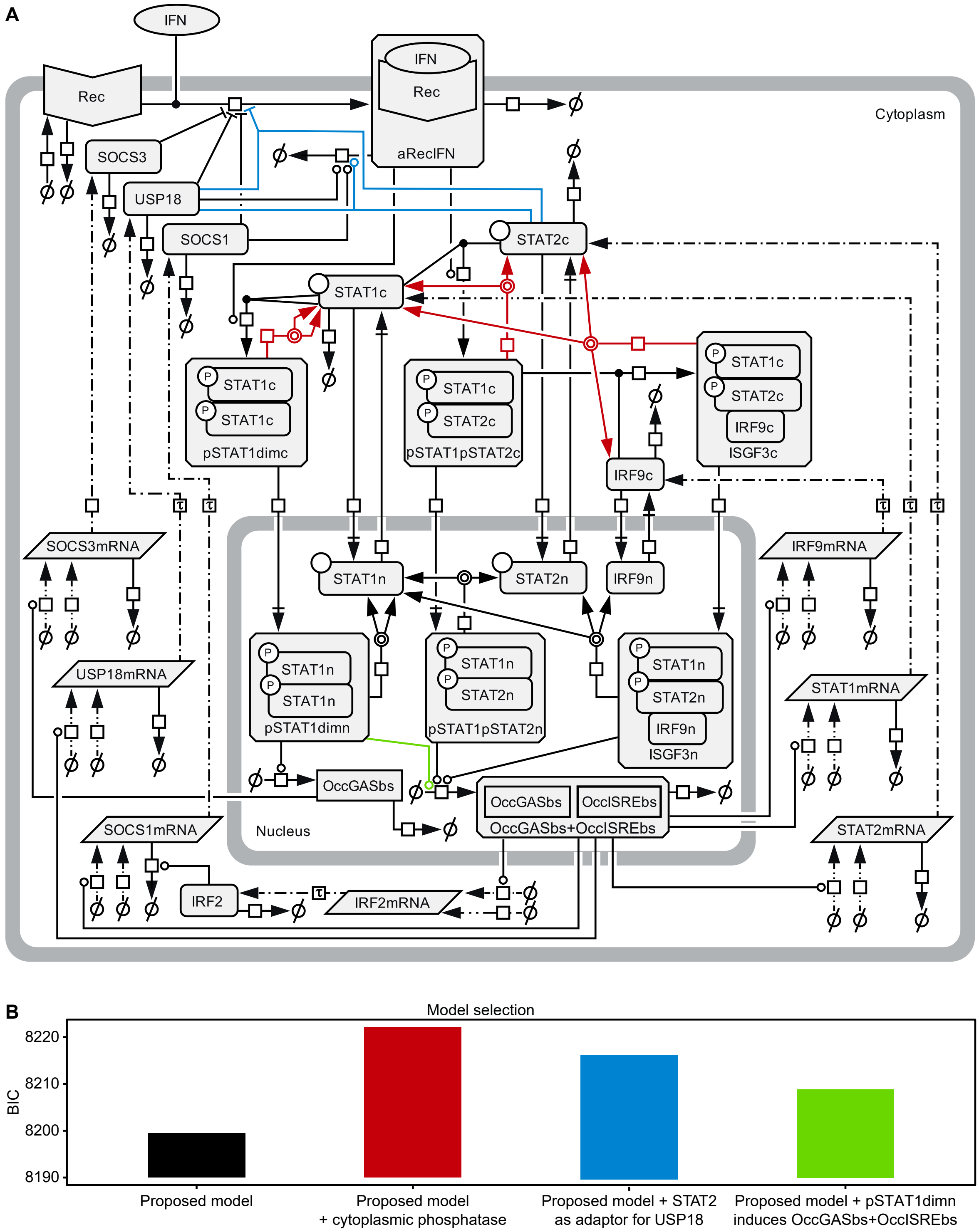

Appendix Figure S5: Model selection based on additional mechanisms
A. The model structure is represented by a process diagram displayed according to Systems Biology Graphical Notation (Le Novere, 2015). Three additional hypothetical mechanisms were tested: A cytoplasmic phosphatase dissociating pSTAT1dimc, pSTAT1pSTAT2c and ISGF3c (red), STAT2 functioning as an adapter for USP18 (blue) and pSTAT1dimn inducing OccGASbs+OccISREbs (green). B. Parameters for each of this three hypothesis were re-estimated based on the experimental data. The Bayesian information criterion (BIC) was calculated, rejecting each additional mechanism.

Appendix Figure S6

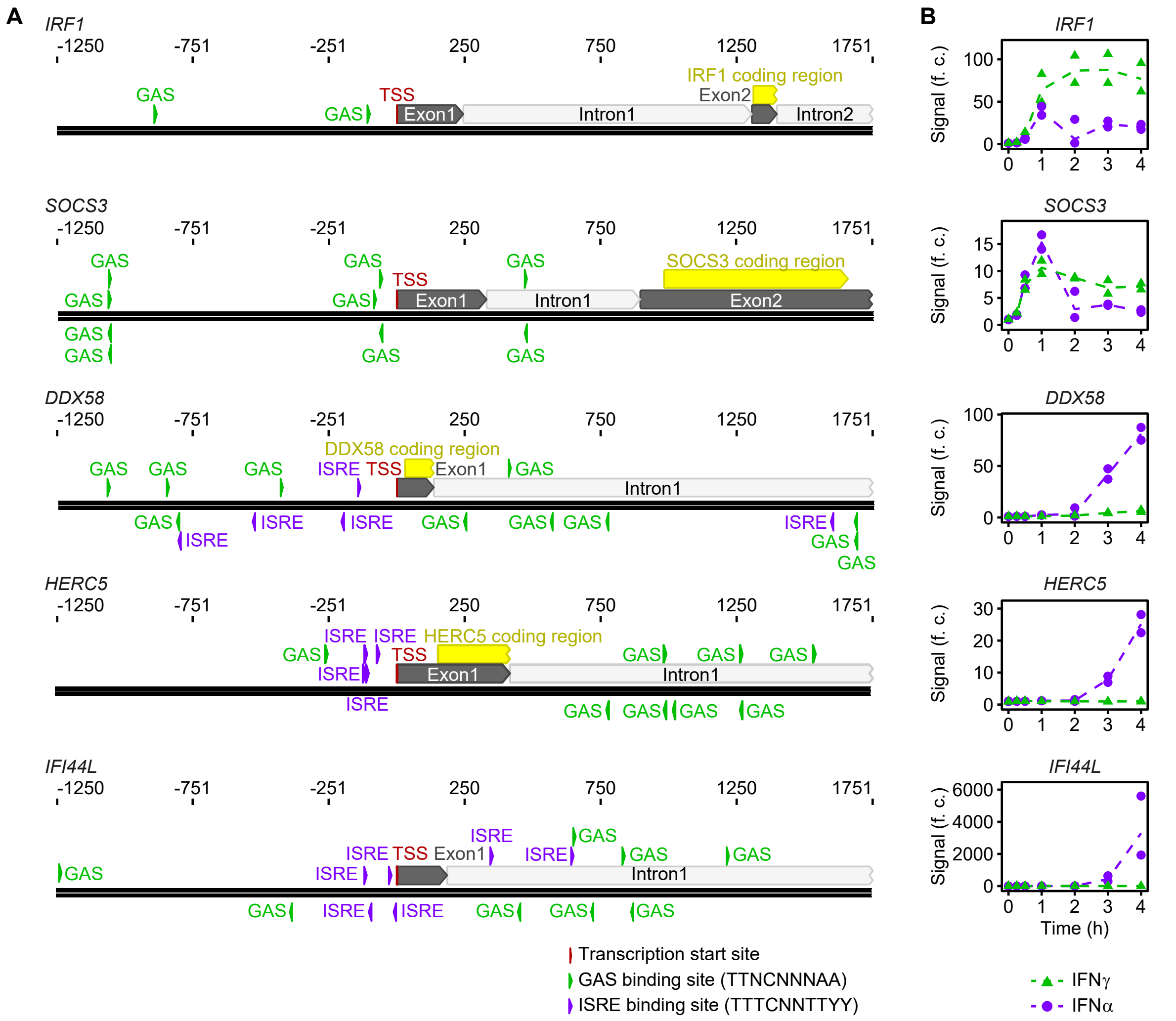

Appendix Figure S6: Identification of GAS- and ISRE-driven interferon target genes
A. Promoter analysis of the human genes *IRF1*, *SOCS3*, *DDX58*, *HERC5* and *IFI44L* was performed of a 3000 bp region around the gene. The following patterns were searched and displayed: TTNCNNNAA (GAS), TTTCNNTTYY (ISRE). Additionally, transcription start site, exons, introns and coding region of the corresponding gene are indicated if present in the analyzed region.
B. Growth factor-depleted Huh7.5 cells were stimulated with either 1400 pM (corresponding to 5000 IU/ml) IFNα or 5000 IU/ml IFNγ for up to four hours and lysed at indicated time points. Interferon-induced expression of target genes was measured by qRT-PCR. RNA levels were normalized to the geometric mean of reference genes *GAPDH*, *HPRT* and *TBP* and were displayed as fold change (f. c.) compared to time point 0. Data from two biological replicates were scaled using a combined scaling and error model (filled symbols), mean values estimated on logarithmic scale are connected with dashed lines.

Appendix Figure S7

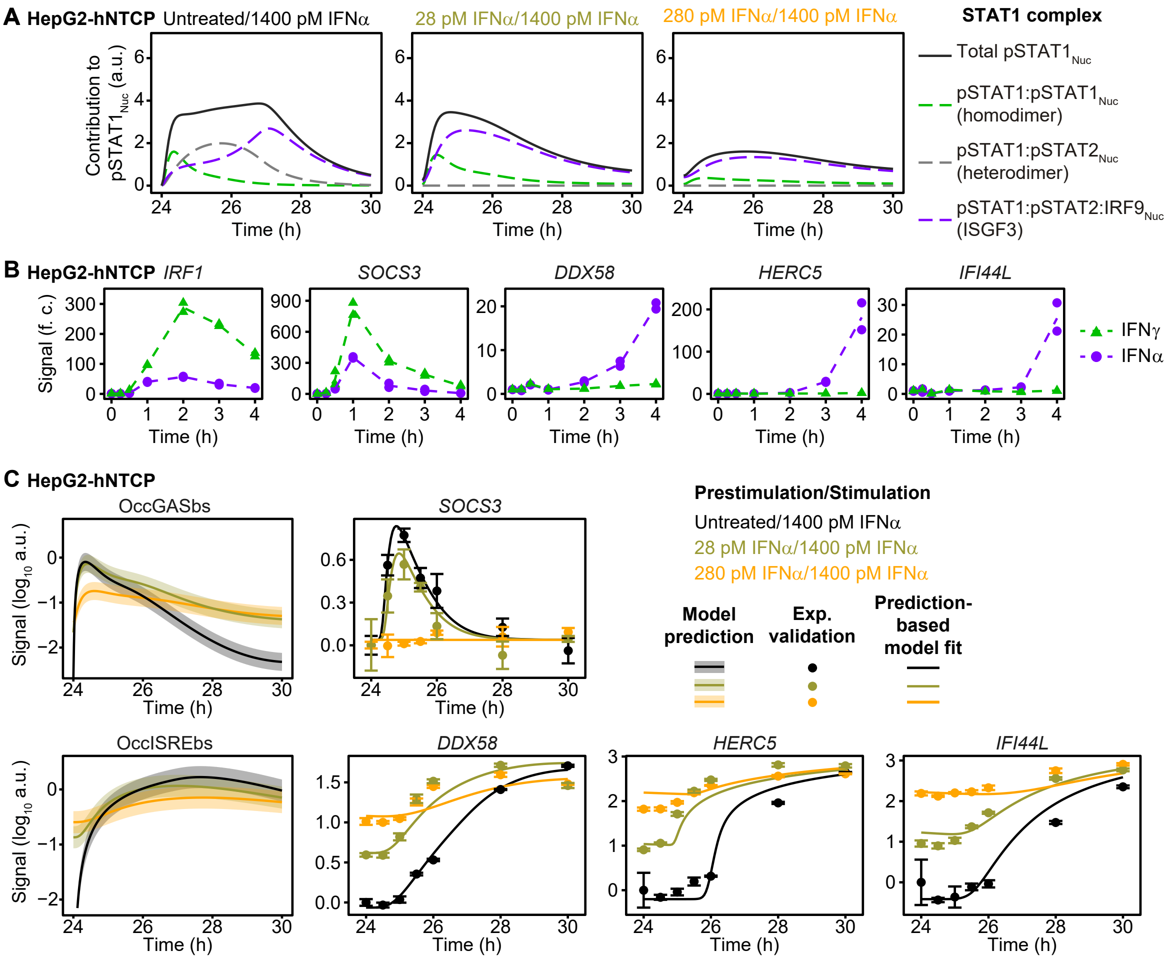

Appendix Figure S7: Model analysis and validation of the dynamics of pSTAT1 complex formation in HepG2-hNTCP cells

A. Model analysis reveals impact of different prestimulation doses on the dynamics of pSTAT1-containing nuclear complexes in HepG2-hNTCP cells. The time-resolved amounts of nuclear pSTAT1 homodimers, pSTAT1:pSTAT2 heterodimers and pSTAT1:pSTAT2:IRF9 trimers were calculated by the mathematical model. Simulations were performed for HepG2-hNTCP cells stimulated with 1400 pM IFNα that were either untreatead or prestimulated with 28 pM IFNα or 280 pM IFNα for 24 hours. Different STAT1 comprising transcription factor complexes are indicated.

B. Growth factor-depleted HepG2-hNTCP cells were stimulated with either 1400 pM (corresponding to 5000 IU/ml) IFNα or 5000 IU/ml IFNγ for up to four hours and lysed at indicated time points. Interferon-induced expression of target genes was measured by qRT-PCR. RNA levels were normalized to the geometric mean of reference genes *GAPDH*, *HPRT* and *TBP* and were displayed as fold change (f. c.) compared to time point 0. Data from two biological replicates were scaled using a combined scaling and error model (filled symbols), mean values estimated on logarithmic scale are connected with dashed lines.

Appendix Figure S7: Model analysis and validation of the dynamics of pSTAT1 complex formation in HepG2-hNTCP cells (Continued)

C. Model predictions of IFNα-induced dynamics of occupied GAS bindings sites (OccGASbs) and of occupied ISRE binding sites (OccISREbs) in HepG2-hNTCP cells without prestimulation and in cells prestimulated for 24 hours with 28 pM and 280 pM IFNα that were subsequently stimulated with 1400 pM IFNα. Model predictions were performed using the prediction profile likelihood method. Lines with shading represent model predictions with 68% confidence intervals. For experimental validation, growth factor-depleted HepG2-hNTCP cells were prestimulated with 0 pM, 28 pM and 280 pM IFNα. After 24 hours cells were stimulated with 1400 pM IFNα and IFNα-induced expression of target genes was measured by qRT-PCR. RNA levels were normalized to the geometric mean of reference genes *GAPDH*, *HPRT* and *TBP*, averaged and displayed as fold change, represented by filled circles with errors representing standard error of the mean calculated from biological replicates (N=3). Except for gene-specific parameters (mRNA synthesis and degradation rates, time-delay parameter and Hill coefficient), qRT-PCR data were used for model validation but not for model calibration.

Appendix Figure S8

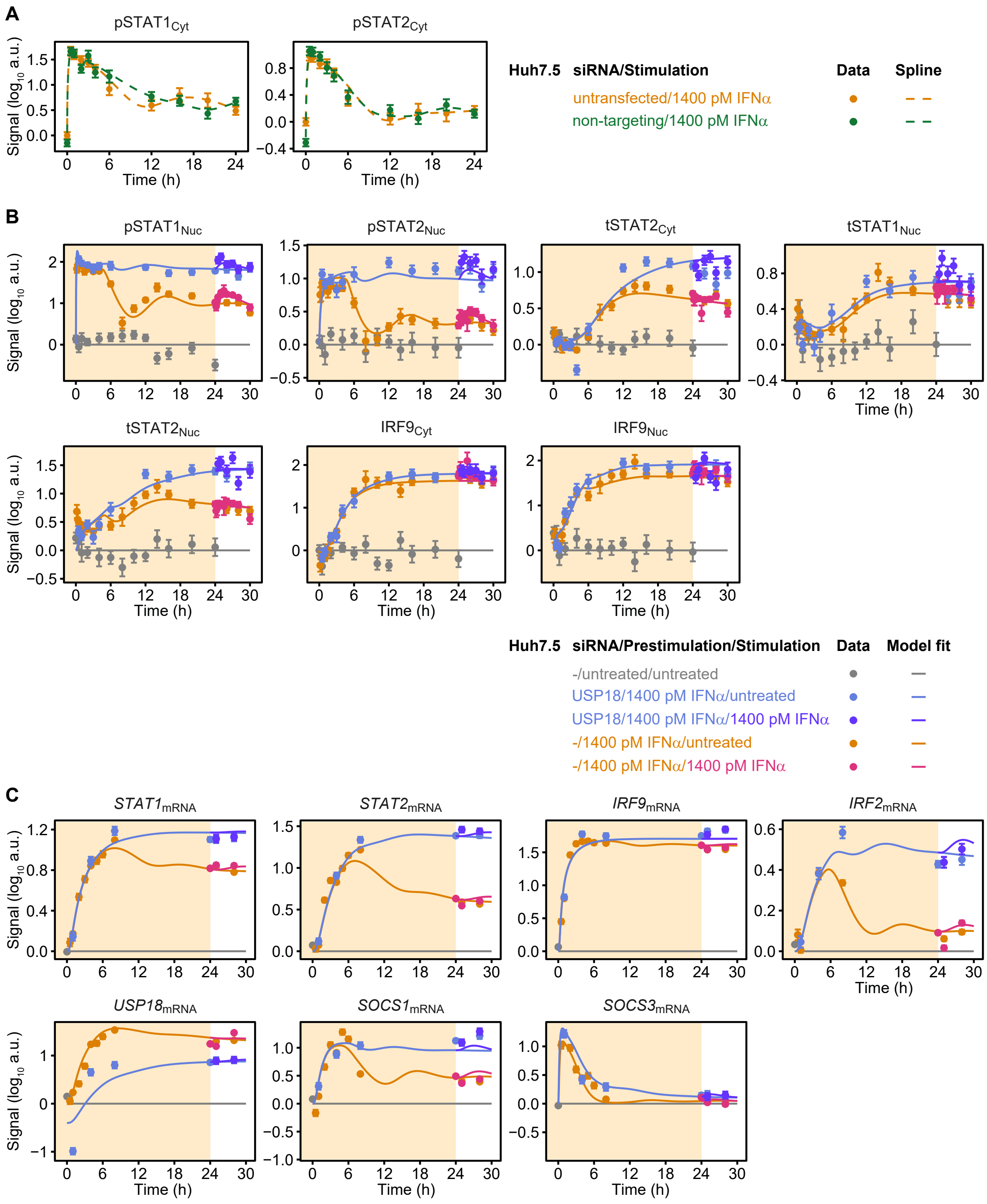

Appendix Figure S8: IFNα-induced signal transduction in USP18 siRNA transfected Huh7.5 cells

A. Untransfected Huh7.5 cells and cells transfected with non-targeting siRNA were stimulated with 1400 pM IFNα for 24 hours. Cytoplasmic lysates were subjected to quantitative immunoblotting. IFNα-induced phosphorylation of STAT1 and STAT2 was detected with chemiluminescence utilizing a CCD camera device (ImageQuant). Data is represented by filled circles with 1σ confidence intervals estimated from biological replicates (N=2 to N=3) using a combined scaling and error model. Dashed lines represent smoothing splines.

Appendix Figure S8: IFNα-induced signal transduction in USP18 siRNA transfected Huh7.5 cells (Continued)

B. Model fit and experimental protein data of Huh7.5 cells transfected with control siRNA or USP18 siRNA are shown. Cells were growth factor-depleted and prestimulated with 1400 pM IFNα (yellow background) and stimulated with 1400 pM IFNα at 24 hours or untreated (white background). IFNα-induced phosphorylation of STAT1 and STAT2 and induction of feedback proteins was detected with chemiluminescence utilizing a CCD camera device (ImageQuant). For modeling purposes data in control siRNA and untransfected Huh7.5 are combined to one condition. Data from multiple time courses scaled together are displayed as filled circles with errors representing 1σ confidence interval estimated from biological replicates (N=2 to N=10) using a combined scaling and error model. Lines represent model trajectories.

C. Model fit and experimental mRNA data of Huh7.5 cells transfected with control siRNA or USP18 siRNA are shown. Cells were growth factor-depleted and prestimulated with 1400 pM IFNα (yellow background) and stimulated with 1400 pM IFNα at 24 hours or untreated (white background). IFNα-induced expression of target genes was measured by qRTPCR. RNA levels were normalized to the geometric mean of reference genes GAPDH, HPRT and TBP and were displayed as fold change, visualized by filled circles with errors representing 1σ confidence-interval estimated from biological replicates (N=3) using a combined scaling and error model. Model trajectories are represented by lines.

Appendix Figure S9

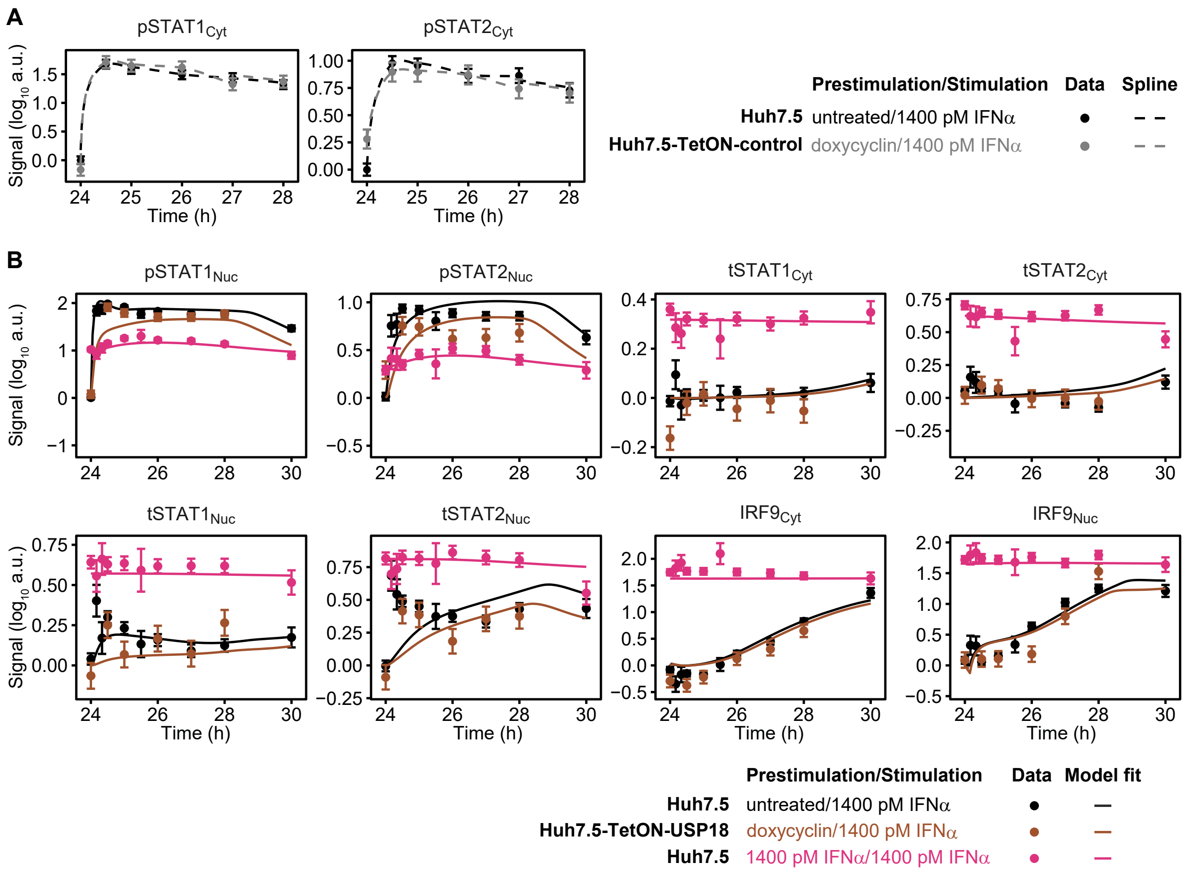

Appendix Figure S9: IFNα-induced signal transduction in USP18 overexpressing Huh7.5 cells

A. Parental Huh7.5 cells and Huh7.5-TetON-control cells treated with doxycycline for 24 hours were stimulated with 1400 pM IFNα. Cytoplasmic lysates were subjected to quantitative immunoblotting. IFNα-induced phosphorylation of STAT1 and STAT2 was detected with chemiluminescence utilizing a CCD camera device (ImageqQant). Data is represented by filled circles with 1σ confidence intervals estimated from biological replicates (N=2 to N=3) using a combined scaling and error model. Dashed lines represent smoothing splines.

B. Model fits and experimental data of Huh7.5-Teton-USP18 treated with doxycycline for 24 hours and stimulated with 1400 pM IFNα or parental Huh7.5 cells prestimulated with 0 or 1400 pM IFNα and stimulated with 1400 pM IFNα after 24 hours are shown. IFNα-induced phosphorylation of STAT1 and STAT2 and induction of feedback proteins was detected with chemiluminescence utilizing a CCD camera device (ImageQuant). For modeling purposes data from Huh7.5-TetON empty vector control and untransduced Huh7.5 are combined to one condition. Data from multiple time courses scaled together are displayed as filled circles with errors representing 1σ confidence interval estimated from biological replicates (N=3 to N=4) using a combined scaling and error model. Lines represent model trajectories.

Appendix Figure S10

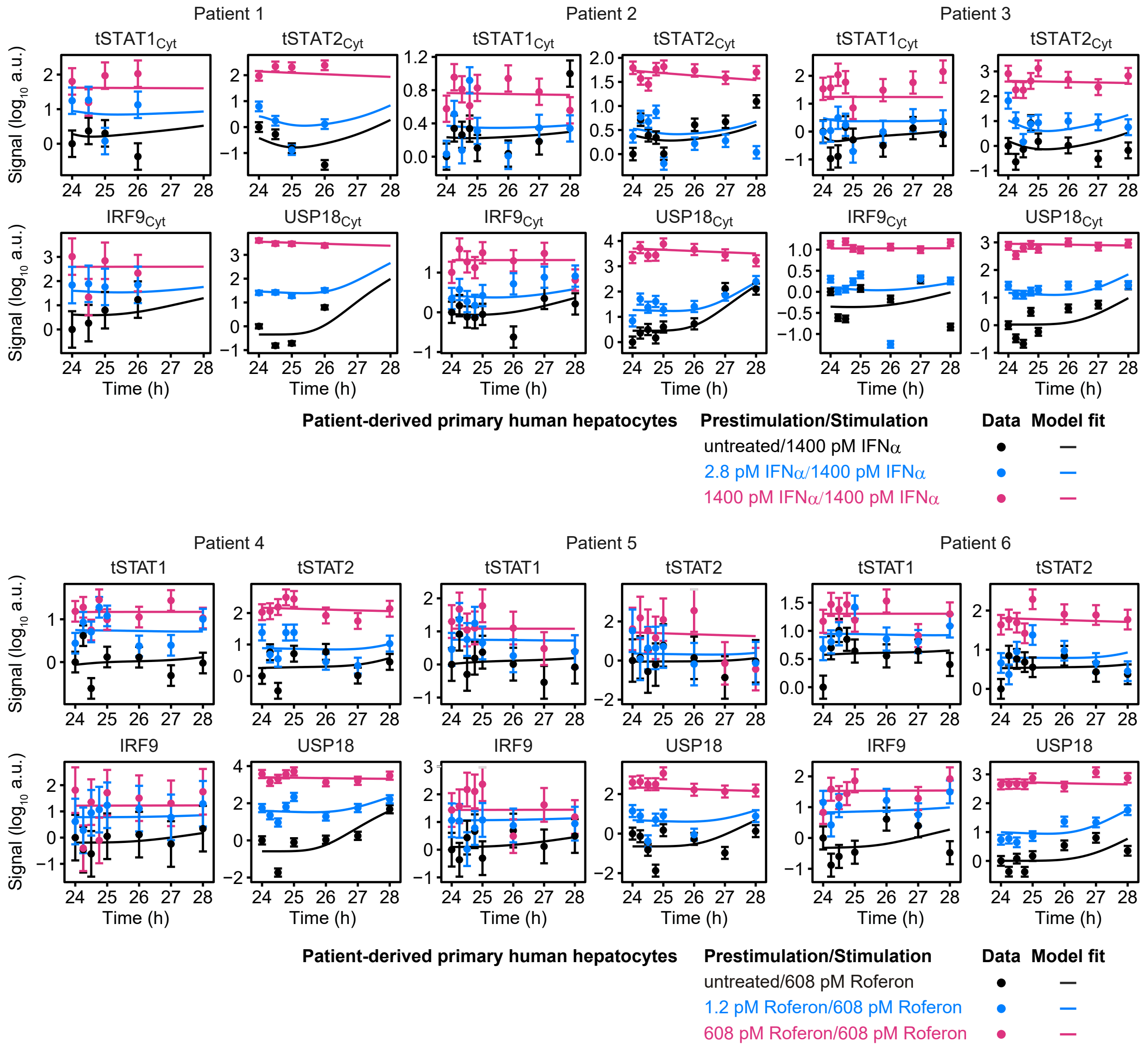

Appendix Figure S10: IFNα-induced signal transduction in patient-derived primary human hepatocytes

Experimental data and model fit of IFNα- and Roferon-induced phosphorylation of cytoplasmic or cellular STAT1 and STAT2 in growth factor-depleted primary human hepatocytes prestimulated with 0, 2.8 or 1400 pM IFNα (patient 1-3) or 0, 1.2 or 608 pM Roferon (patient 4-6). Experimental data is represented by filled circles (N=1 per patient). Experimental errors were estimated from the signal variance of the hepatocytes prestimulated with 1400 pM IFNα. Lines indicate model fits.

Appendix Figure S11

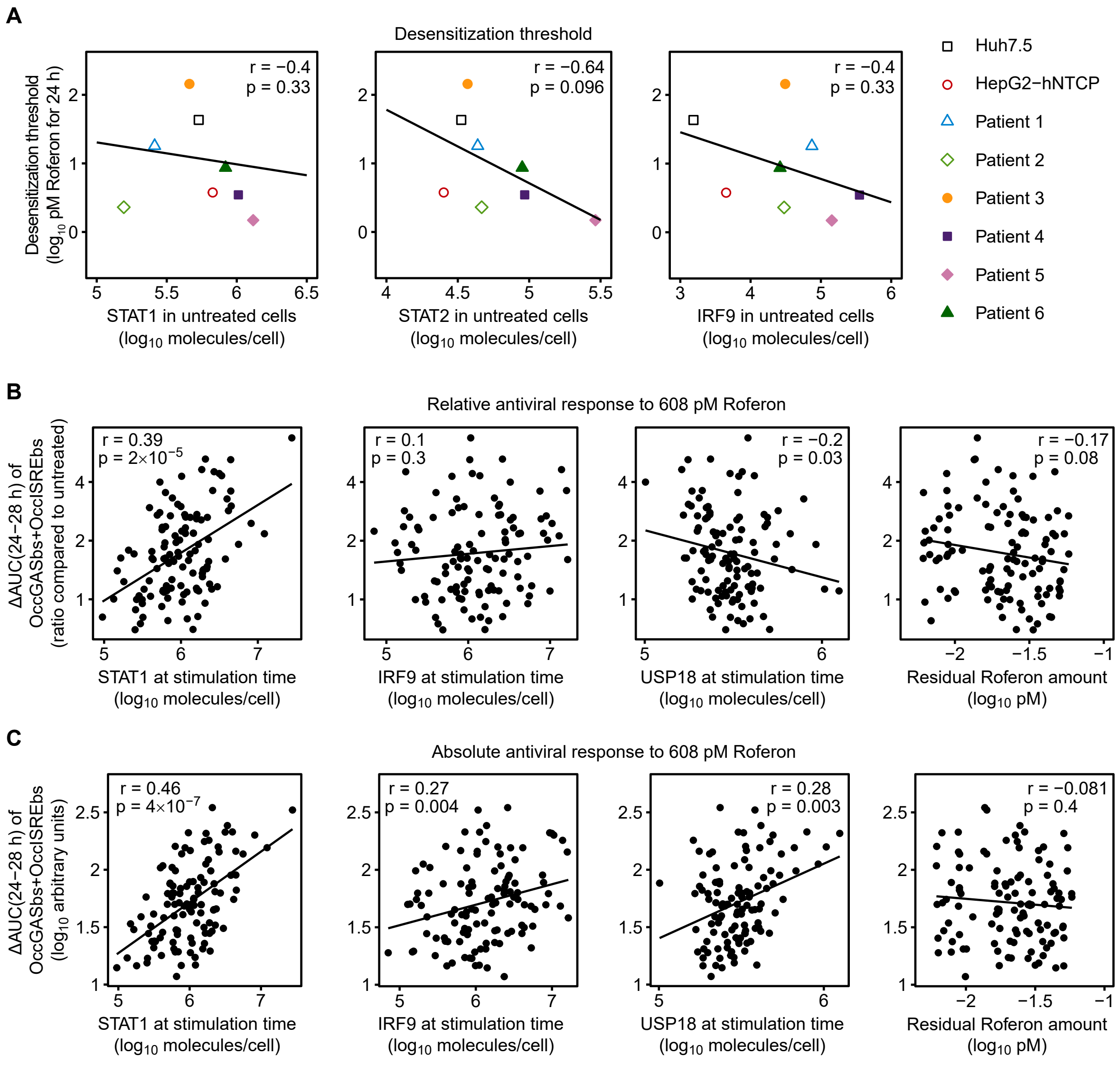

Appendix Figure S11: Correlations of desensitization threshold and antiviral response with protein abundances

A. Desensitization thresholds for Huh7.5 and HepG2-hNTCP cells and patient-derived primary human hepatocytes determined in Fig. 7A are plotted against the amount of STAT1, STAT2 and IRF9 in corresponding untreated cells. Spearman's rank order correlation coefficient (r) and p-value (p) are indicated.

B. For each patient in the virtual patient cohort defined in Fig. 7C a stimulation with 608 pM Roferon was simulated and the relative antiviral response (ΔAUC(24 – 28 h) of OccGASbs+OccISREbs compared to untreated) was calculated. The relative antiviral response was plotted against the cellular abundance of STAT1, IRF9, USP18 and the residual Roferon amount. Spearman's rank order correlation coefficient (r) and p-value (p) are indicated. C. For each patient in the virtual patient cohort a stimulation with 608 pM Roferon was simulated and the absolute antiviral response (ΔAUC(24 – 28 h) of OccGASbs+OccISREbs) was calculated. The absolute antiviral response was plotted against the cellular abundance of STAT1, IRF9, USP18 and the residual Roferon amount. Spearman's rank order correlation coefficient (r) and p-value (p) are indicated.

Appendix Figure S12

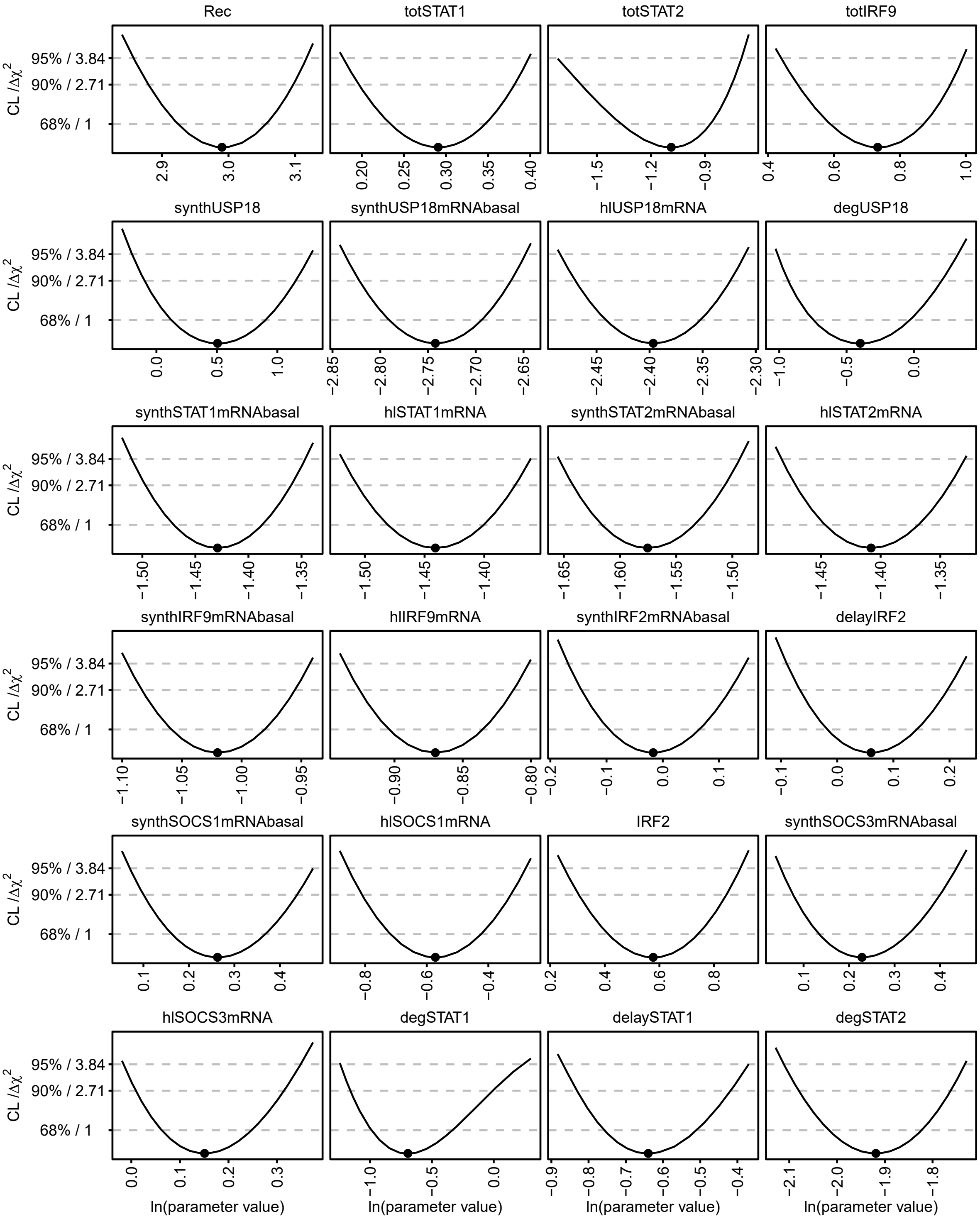

Appendix Figure S12 : Profile likelihood of model parameters

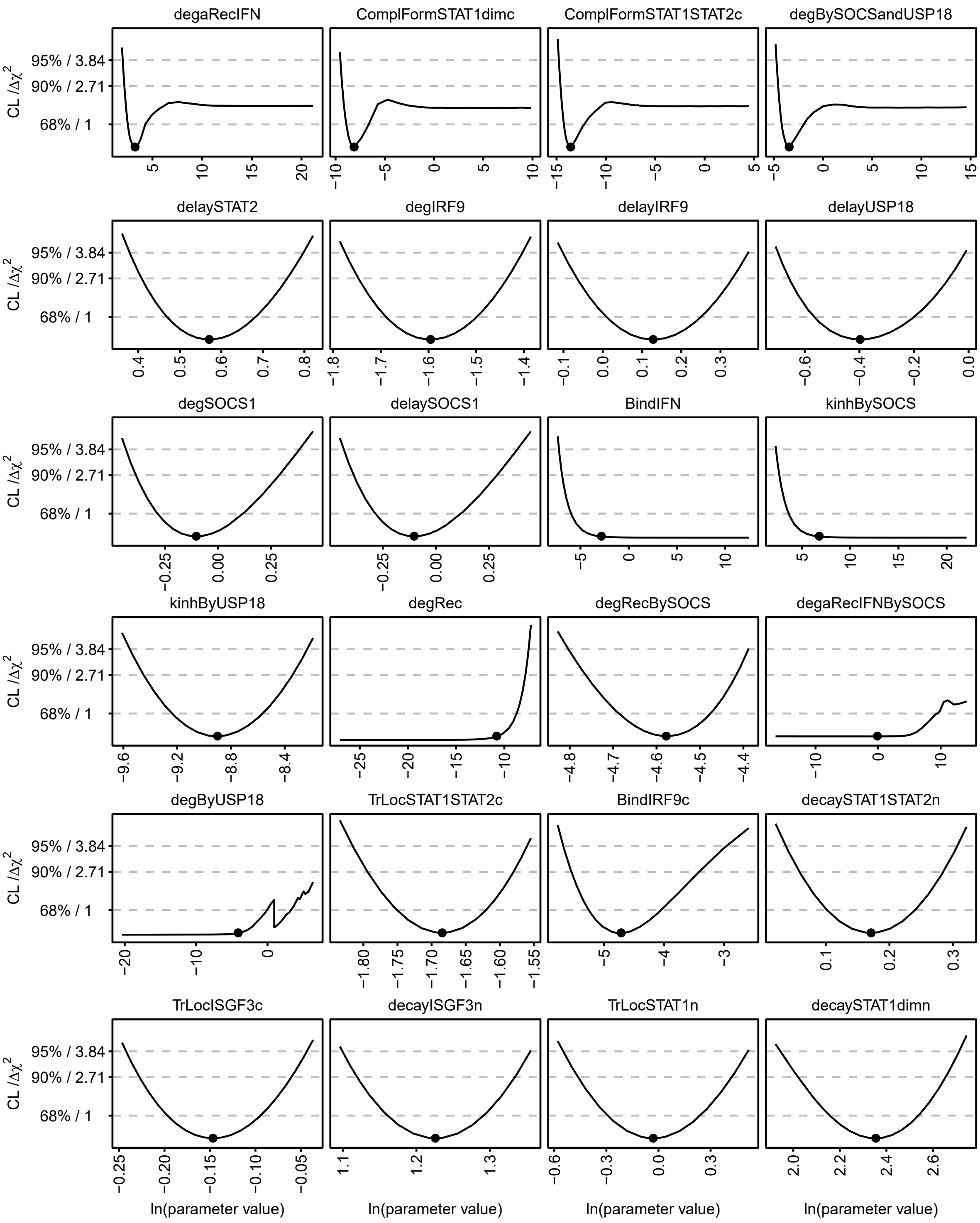

Appendix Figure S12: Profile likelihood of model parameters (Continued)

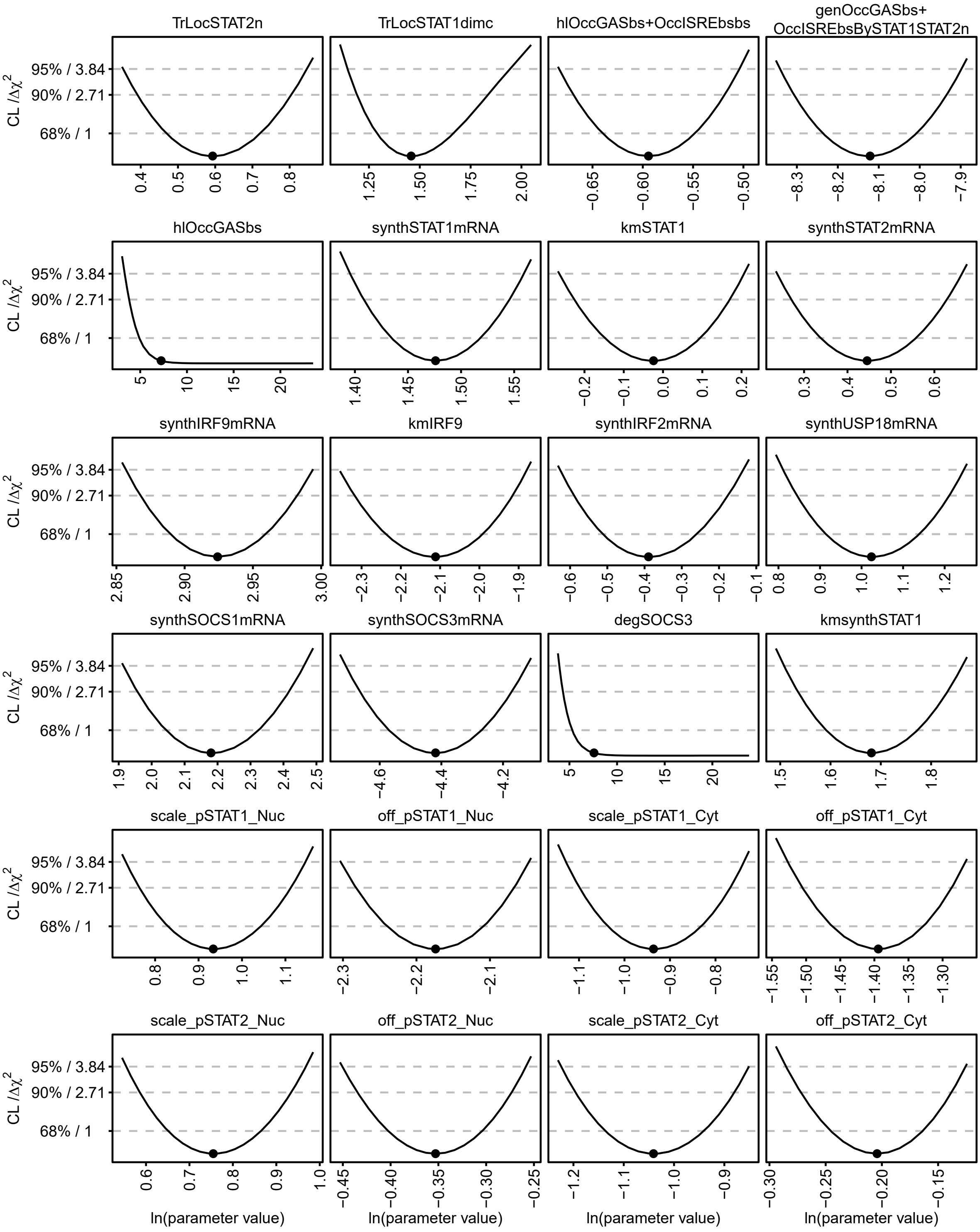

Appendix Figure S12: Profile likelihood of model parameters (Continued)

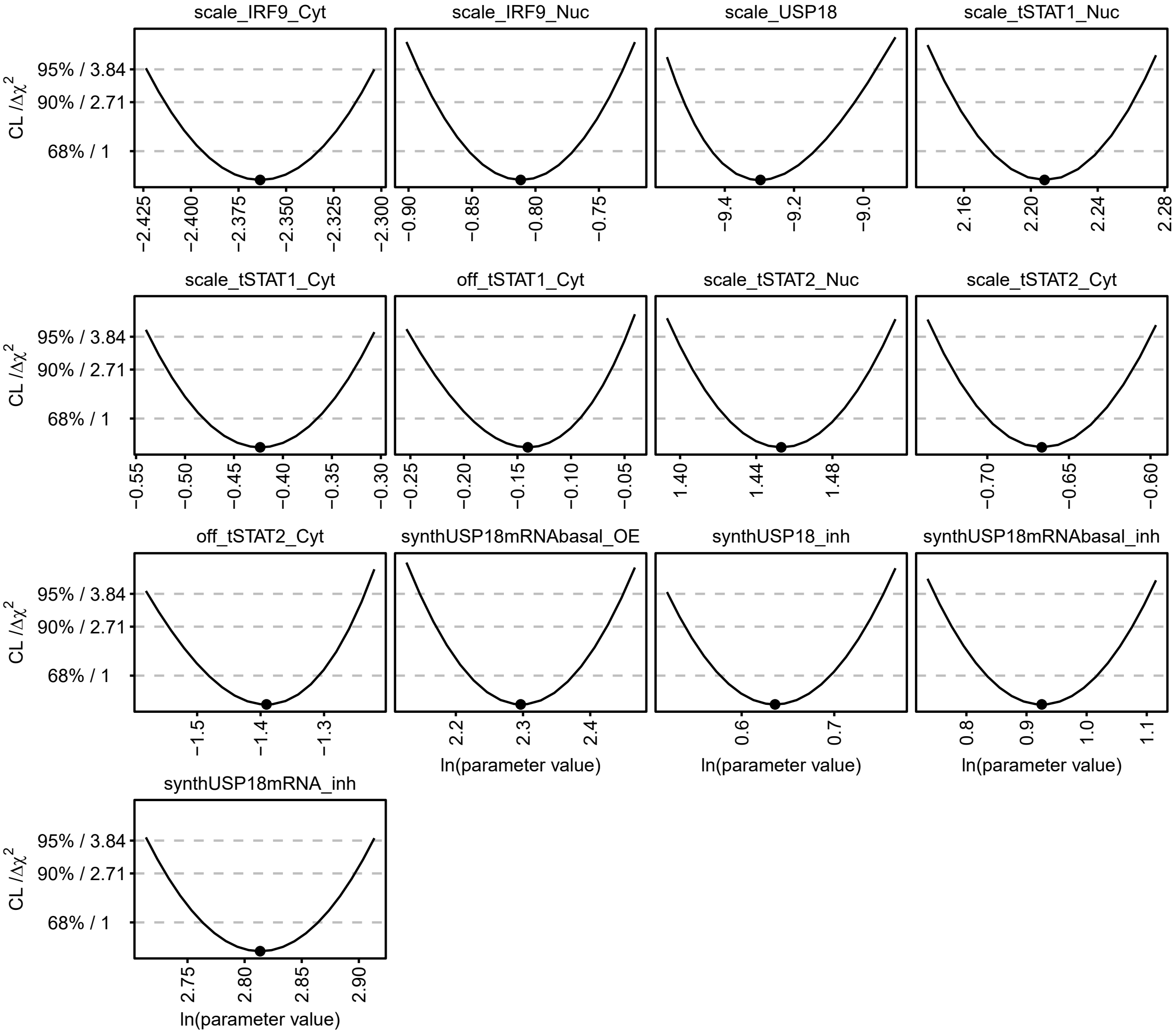

Appendix Figure S12: Profile likelihood of model parameters (Continued)
The profile likelihood (Raue et al., 2009) was calculated for each parameter, which systematically trace an optimal path over the likelihood to determine parameter confidence bounds. If the parameter is identifiable, the negative log-likelihood will reach a statistical threshold in both directions. If the negative log-likelihood does not reach this bound on either side, then it is not possible to determine a range for the parameter and the parameter is classified as non-identifiable. The solid lines indicate the profile likelihood. The dashed lines indicate the threshold to assess confidence intervals. Filled circles indicate the optimal parameter values.

Appendix Figure S13

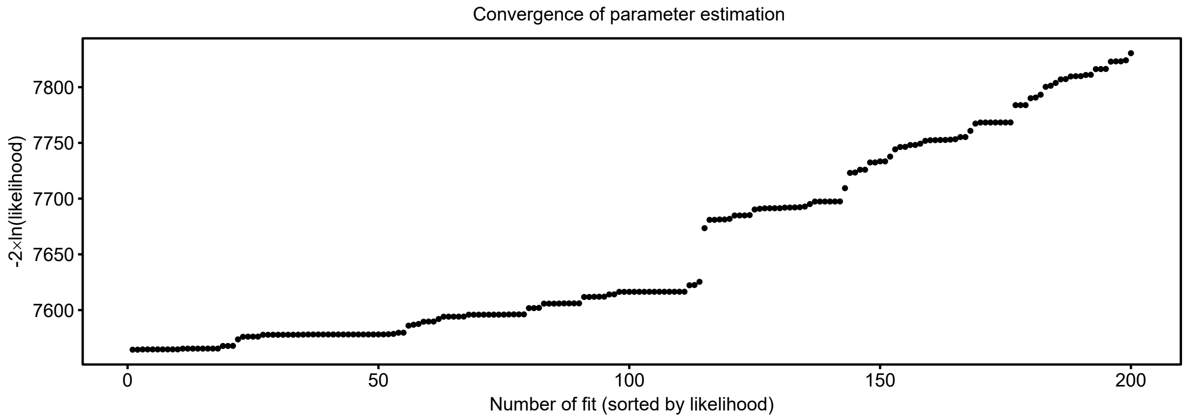

Appendix Figure S13: Assessment of the optimization performance by a waterfall plot The results of the 200 best out of 1000 optimization runs starting from randomly selected parameter sets were displayed sorted by their objective value (Raue et al., 2013). The global optimum was found in 18 of the 200 cases.

Tables

Appendix Table S1

Educt	→	Product	Rate	Description	Justification
	→	Rec	prodRec	Receptor production	(Novick et al., 1994)
Rec	→		degRec · Rec	Receptor degradation basal	(Bhattacharya et al., 2010)
Rec	→		degRecBySOCS · Rec · SOCS1	Receptor degradation by SOCS1	(Piganis et al., 2011)
IFN + Rec	→	aRecIFN	$\mathrm{BindIFN} \cdot \mathrm{IFN} \cdot \mathrm{Rec} \cdot \frac{1}{\mathrm{SOCS3}} \cdot \frac{1}{1+\mathrm{kinhBySOCS}\cdot\mathrm{SOCS1}} \cdot \frac{1}{1+\mathrm{kinhByUSP18}\cdot\mathrm{USP18}\cdot(1+\mathrm{kinhBySTAT2}\cdot\mathrm{STAT2})}$	Active receptor complex formation	(Chen et al., 2000; Malakhova et al., 2006) STAT2 influence rejected based on our results (Figure S5B)
aRecIFN	→		aRecIFN · degaRecIFN	Active receptor complex degradation basal	(Kumar et al., 2003)
aRecIFN	→		aRecIFN · degaRecIFNBySOCS · SOCS1	Active receptor complex degradation by SOCS1	(Piganis et al., 2011)
aRecIFN	→		aRecIFN · degByUSP18 · USP18	Active receptor complex degradation by USP18	Rejected based on our results (Figure 5C)
aRecIFN	→		aRecIFN · degByUSP18andSTAT2 · USP18 · STAT2	Active receptor complex degradation by USP18 and STAT2 as adaptor for USP18	Rejected based on our results (Figure S5B)
aRecIFN	→		aRecIFN · degBySOCSandUSP18 · SOCS1 · USP18	Active receptor complex degradation by USP18 and SOCS1 (synergy)	Included based on our results (Figure 5C)
aRecIFN	→		aRecIFN · degBySOCSandUSP18andSTAT2 · SOCS1 · USP18 · STAT2	Active receptor complex degradation by USP18 and SOCS1 (synergy) and STAT2 as adaptor for USP18	Rejected based on our results (Figure S5B)
USP18	→		degUSP18 · USP18	USP18 degradation	(Zhang et al., 2015)
STAT1c + STAT2c	→	pSTAT1pSTAT2c	ComplFormSTAT1STAT2c · aRecIFN · STAT1c · STAT2c	Heterodimer complex formation	(Li et al., 1996)
pSTAT1pSTAT2c	→	pSTAT1pSTAT2n	TrLocSTAT1STAT2c · pSTAT1pSTAT2c	Heterodimer translocation to nucleus	(Banninger and Reich, 2004)
pSTAT1pSTAT2c	→	STAT1c + STAT2c	decaySTAT1STAT2c · pSTAT1pSTAT2c	Heterodimer decay	Rejected based on our results (Figure S5B)
pSTAT1pSTAT2n	→	STAT1n + STAT2n	decaySTAT1STAT2n · pSTAT1pSTAT2n	Heterodimer decay	(Banninger and Reich, 2004)
pSTAT1pSTAT2c + IRF9c	→	ISGF3c	BindIRF9c · pSTAT1pSTAT2c · IRF9c	ISGF3 complex formation	(Platanias, 2005)
ISGF3c	→	ISGF3n	TrLocISGF3c · ISGF3c	ISGF3 transloction to nucleus	(Schindler et al., 1992)

Educt	→	Product	Rate	Description	Justification
ISGF3c	→	STAT1c + STAT2c + IRF9c	decayISGF3c · ISGF3c	ISGF3 decay	Rejected based on our results (Figure S5B)
ISGF3n	→	STAT1n + STAT2n + IRF9n	decayISGF3n · ISGF3n	ISGF3 decay	(Banninger and Reich, 2004)
2·STAT1c	→	pSTAT1dimc	ComplFormSTAT1dimc · aRecIFN · STAT1c · STAT1c	Homodimer complex formation	(Decker et al., 1991)
pSTAT1dimc	→	pSTAT1dimn	TrLocSTAT1dimc · pSTAT1dimc	Homodimer translocation to nucleus	(Banninger and Reich, 2004)
pSTAT1dimc	→	2·STAT1c	decaySTAT1dimn · pSTAT1dimc	Homodimer decay	Rejected based on our results (Figure S5B)
pSTAT1dimn	→	2·STAT1n	decaySTAT1dimc · pSTAT1dimn	Homodimer decay	(Mertens et al., 2006)
	→	OccGASbs+ OccISREbs	genOccGASbs+OccISREbsByISGF3n · ISGF3n	OccGASbs+OccISREbs formation by ISGF3	(Schindler et al., 1992)
	→	OccGASbs+ OccISREbs	genOccGASbs+OccISREbsBySTAT1STAT2n · pSTAT1pSTAT2n	OccGASbs+OccISREbs formation by heterodimer	(Banninger and Reich, 2004)
	→	OccGASbs+ OccISREbs	OccGASbs+OccISREbsBySTAT1dimn · pSTAT1dimn	OccGASbs+OccISREbs formation by homodimer	Rejected based on our results (Figure S5B)
OccGASbs+ OccISREbs	→		hlOccGASbs+OccISREbs · OccGASbs+OccISREbs	OccGASbs+OccISREbs decay	(Harada et al., 1996)
	→	OccGASbs	genOccGASbsBySTAT1dimn · pSTAT1dimn	OccGASbs site formation	(Decker et al., 1991)
OccGASbs	→		hlOccGASbs · OccGASbs	OccGASbs decay	(Decker et al., 1991)
STAT1c	→		degSTAT1 · STAT1c	STAT1 degradation	(Shuai and Liu, 2003)
STAT1n	→	STAT1c	TrLocSTAT1n · STAT1n	STAT1 transloction to cytoplasm	(Meyer et al., 2002)
STAT1c	→	STAT1n	TrLocSTAT1c · STAT1c	STAT1 transloction to nucleus	(Meyer et al., 2002)
STAT2c	→		degSTAT2 · STAT2c	STAT2 degradation	(Shuai and Liu, 2003)
STAT2n	→	STAT2c	TrLocSTAT2n · STAT2n	STAT2 transloction to cytoplasm	(Banninger and Reich, 2004)
STAT2c	→	STAT2n	TrLocSTAT2c · STAT2c	STAT2 transloction to nucleus	(Banninger and Reich, 2004)
IRF9n	→	IRF9c	TrLocIRF9n · IRF9n	IRF9 transloction to cytoplasm	(Lau et al., 2000)
IRF9c	→	IRF9n	TrLocIRF9c · IRF9c	IRF9 transloction to nucleus	(Lau et al., 2000)
IRF9c	→		degIRF9 · IRF9c	IRF9 degradation	(Paul et al., 2018)
SOCS1	→		degSOCS1 · SOCS1	SOCS1 degradation	(Siewert et al., 1999)
	→	SOCS3	synthSOCS3 · SOCS3mRNA	SOCS3 synthesis	(Ilangumaran et al., 2004)
SOCS3	→		degSOCS3 · SOCS3	SOCS3 degradation	(Siewert et al., 1999)
	→	STAT1mRNA	synthSTAT1mRNAbasal	STAT1mRNA basal production	(Lehtonen et al., 1997)

**Educt**	→	**Product**	**Rate**	**Description**	**Justification**
	→	STAT1mRNA	synthSTAT1mRNA · OccGASbs+OccISREbs · $\frac{1}{\text{kmSTAT1}+\text{OccGASbs+OccISREbs}}$	STAT1mRNA production by GAS+ISRE	(Lehtonen et al., 1997)
STAT1mRNA	→		hlSTAT1mRNA · STAT1mRNA	STAT1mRNA decay	(Lehtonen et al., 1997)
	→	STAT2mRNA	synthSTAT2mRNAbasal	STAT2mRNA basal production	(Lehtonen et al., 1997)
	→	STAT2mRNA	synthSTAT2mRNA · OccGASbs+OccISREbs	STAT2mRNA production by GAS+ISRE	(Lehtonen et al., 1997)
STAT2mRNA	→		hlSTAT2mRNA · STAT2mRNA	STAT2mRNA decay	(Lehtonen et al., 1997)
	→	IRF9mRNA	synthIRF9mRNAbasal	IRF9mRNA basal production	(Lehtonen et al., 1997)
	→	IRF9mRNA	synthIRF9mRNA · OccGASbs+OccISREbs · $\frac{1}{\text{kmIRF9}+\text{OccGASbs+OccISREbs}}$	IRF9mRNA production by GAS+ISRE	(Lehtonen et al., 1997)
IRF9mRNA	→		hlIRF9mRNA · IRF9mRNA	IRF9mRNA decay	(Lehtonen et al., 1997)
	→	IRF2mRNA	synthIRF2mRNAbasal	IRF2mRNA basal production	(Taniguchi et al., 2001)
	→	IRF2mRNA	synthIRF2mRNA · OccGASbs+OccISREbs	IRF2mRNA production by GAS+ISRE	(Harada et al., 1989)
IRF2mRNA	→	IRF2	delayIRF2 · IRF2mRNA	IRF2 translation and decay of mRNA	(Taniguchi et al., 2001)
	→	USP18mRNA	synthUSP18mRNAbasal	USP18mRNA basal production	(Sarasin-Filipowicz et al., 2009)
	→	USP18mRNA	synthUSP18mRNA · OccGASbs+OccISREbs	USP18mRNA production by GAS+ISRE	(Malakhova et al., 2006)
USP18mRNA	→		hlUSP18mRNA · USP18mRNA	USP18mRNA decay	(Sarasin-Filipowicz et al., 2009)
	→	SOCS1mRNA	synthSOCS1mRNAbasal	SOCS1mRNA basal production	(Ilangumaran et al., 2004)
	→	SOCS1mRNA	synthSOCS1mRNA · OccGASbs+OccISREbs	SOCS1mRNA production by GAS+ISRE	(Song and Shuai, 1998)
SOCS1mRNA	→		hlSOCS1mRNA · SOCS1mRNA · (1 + IRF2)	SOCS1mRNA decay	(Harada et al., 1989)
	→	SOCS3mRNA	synthSOCS3mRNAbasal	SOCS3mRNA basal production	(Ilangumaran et al., 2004)
	→	SOCS3mRNA	synthSOCS3mRNA · OccGASbs	SOCS3mRNA production by GAS	(Song and Shuai, 1998)
SOCS3mRNA	→		hlSOCS3mRNA · SOCS3mRNA	SOCS3mRNA decay	(Ehlting et al., 2007)
IRF2	→		degIRF2 · IRF2	IRF2 degradation	(Taniguchi et al., 2001)
	→	STAT1_LC_1	synthSTAT1 · STAT1mRNA · $\frac{1}{\text{kmsynthSTAT1}+\text{STAT1mRNA}}$	Linear chain for time delay in STAT1 translation	(MacDonald, 1976)
STAT1_LC_1	→	STAT1_LC_2	delaySTAT1 · STAT1_LC_1	STAT1 chain step 1	(MacDonald, 1976)
STAT1_LC_2	→	STAT1_LC_3	delaySTAT1 · STAT1_LC_2	STAT1 chain step 2	(MacDonald, 1976)
STAT1_LC_3	→	STAT1c	delaySTAT1 · STAT1_LC_3	STAT1 chain step 3	(MacDonald, 1976)

Educt	→	Product	Rate	Description	Justification
	→	STAT2_LC_1	synthSTAT2 · STAT2mRNA	Linear chain for time delay in STAT2 translation	(MacDonald, 1976)
STAT2_LC_1	→	STAT2_LC_2	delaySTAT2 · STAT2_LC_1	STAT2 chain step 1	(MacDonald, 1976)
STAT2_LC_2	→	STAT2_LC_3	delaySTAT2 · STAT2_LC_2	STAT2 chain step 2	(MacDonald, 1976)
STAT2_LC_3	→	STAT2_LC_4	delaySTAT2 · STAT2_LC_3	STAT2 chain step 3	(MacDonald, 1976)
STAT2_LC_4	→	STAT2_LC_5	delaySTAT2 · STAT2_LC_4	STAT2 chain step 4	(MacDonald, 1976)
STAT2_LC_5	→	STAT2c	delaySTAT2 · STAT2_LC_5	STAT2 chain step 5	(MacDonald, 1976)
	→	IRF9_LC_1	synthIRF9 · IRF9mRNA	Linear chain for time delay in IRF9 translation	(MacDonald, 1976)
IRF9_LC_1	→	IRF9_LC_2	delayIRF9 · IRF9_LC_1	IRF9 chain step 1	(MacDonald, 1976)
IRF9_LC_2	→	IRF9c	delayIRF9 · IRF9_LC_2	IRF9 chain step 2	(MacDonald, 1976)
	→	USP18_LC_1	synthUSP18 · USP18mRNA	Linear chain for time delay in USP18 translation	(MacDonald, 1976)
USP18_LC_1	→	USP18_LC_2	delayUSP18 · USP18_LC_1	USP18 chain step 1	(MacDonald, 1976)
USP18_LC_2	→	USP18	delayUSP18 · USP18_LC_2	USP18 chain step 2	(MacDonald, 1976)
	→	SOCS1_LC_1	synthSOCS1 · SOCS1mRNA	Linear chain for time delay in SOCS1 translation	(MacDonald, 1976)
SOCS1_LC_1	→	SOCS1	delaySOCS1 · SOCS1_LC_1	SOCS1 chain step 1	(MacDonald, 1976)
**Validation model**					
	→	OccISREbs	genOccGASbs+OccISREbsByISGF3n · ISGF3n	OccISREbs formation by ISGF3	(Schindler et al., 1992)
OccISREbs	→		hlOccGASbs+OccISREbs · OccISREbs	OccISREbs decay	(Harada et al., 1996)
	→	IRF1_LC_1	synthIRF1mRNAbasal	IRF1mRNA basal production	Our results (Figure EV3B)
	→	IRF1_LC_1	synthIRF1mRNA · $\frac{\text{OccGASbs}^{\text{n\_IRF1}}}{\text{kmIRF1}^{\text{n\_IRF1}}+\text{OccGASbs}^{\text{n\_IRF1}}}$	IRF1mRNA production with Hill kinetic and linear chain for time delay	Our results (Figure EV3B)
IRF1_LC_1	→	IRF1_LC_2	delayIRF1mRNA · IRF1_LC_1	IRF1 chain step 1	(MacDonald, 1976)
IRF1_LC_2	→	IRF1_LC_3	delayIRF1mRNA · IRF1_LC_2	IRF1 chain step 2	(MacDonald, 1976)
IRF1_LC_3	→	IRF1mRNA	delayIRF1mRNA · IRF1_LC_3	IRF1 chain step 3	(MacDonald, 1976)
IRF1mRNA	→		hlIRF1mRNA · IRF1mRNA	IRF1mRNA decay	Our results (Figure EV3B)
	→	SOCS3_LC_1	synthSOCS3mRNAbasal	SOCS3mRNA basal production (validation model)	Our results (Figure EV3B)
	→	SOCS3_LC_1	synthSOCS3mRNA · $\frac{\text{OccGASbs}^{\text{n\_SOCS3}}}{\text{kmSOCS3}^{\text{n\_SOCS3}}+\text{OccGASbs}^{\text{n\_SOCS3}}}$	SOCS3mRNA production with Hill kinetic and linear chain for time delay (validation model)	Our results (Figure EV3B)
SOCS3_LC_1	→	SOCS3_LC_2	delaySOCS3mRNA · SOCS3_LC_1	SOCS3 chain step 1 (validation model)	(MacDonald, 1976)
SOCS3_LC_2	→	SOCS3_LC_3	delaySOCS3mRNA · SOCS3_LC_2	SOCS3 chain step 2 (validation model)	(MacDonald, 1976)
SOCS3_LC_3	→	SOCS3mRNA	delaySOCS3mRNA · SOCS3_LC_3	SOCS3 chain step 3 (validation model)	(MacDonald, 1976)

Educt	→	Product	Rate	Description	Justification
SOCS3mRNA	→		hlSOCS3mRNA · SOCS3mRNA	SOCS3mRNA decay (validation model)	Our results (Figure EV3B)
	→	DDX58_LC_1	synthDDX58mRNAbasal	DDX58mRNA basal production	Our results (Figure EV3B)
	→	DDX58_LC_1	synthDDX58mRNA · $\frac{\text{OccISREbs}^{\text{n\_DDX58}}}{\text{kmDDX58}^{\text{n\_DDX58}}+\text{OccISREbs}^{\text{n\_DDX58}}}$	DDX58mRNA production with Hill kinetic and linear chain for time delay	Our results (Figure EV3B)
DDX58_LC_1	→	DDX58_LC_2	delayDDX58mRNA · DDX58_LC_1	DDX58 chain step 1	(MacDonald, 1976)
DDX58_LC_2	→	DDX58_LC_3	delayDDX58mRNA · DDX58_LC_2	DDX58 chain step 2	(MacDonald, 1976)
DDX58_LC_3	→	DDX58mRNA	delayDDX58mRNA ·DDX58_LC_3	DDX58 chain step 3	(MacDonald, 1976)
DDX58mRNA	→		hlDDX58mRNA ·DDX58mRNA	DDX58mRNA decay	Our results (Figure EV3B)
	→	HERC5_LC_1	synthHERC5mRNAbasal	HERC5mRNA basal production	Our results (Figure EV3B)
	→	HERC5_LC_1	synthHERC5mRNA · $\frac{\text{OccISREbs}^{\text{n\_HERC5}}}{\text{kmHERC5}^{\text{n\_HERC5}}+\text{OccISREbs}^{\text{n\_HERC5}}}$	HERC5mRNA production with Hill kinetic and linear chain for time delay	Our results (Figure EV3B)
HERC5_LC_1	→	HERC5_LC_2	delayHERC5mRNA · HERC5_LC_1	HERC5 chain step 1	(MacDonald, 1976)
HERC5_LC_2	→	HERC5_LC_3	delayHERC5mRNA · HERC5_LC_2	HERC5 chain step 2	(MacDonald, 1976)
HERC5_LC_3	→	HERC5mRNA	delayHERC5mRNA ·HERC5_LC_3	HERC5 chain step 3	(MacDonald, 1976)
HERC5mRNA	→		hlHERC5mRNA ·HERC5mRNA	HERC5mRNA decay	Our results (Figure EV3B)
	→	IFI44L_LC_1	synthIFI44LmRNAbasal	IFI44LmRNA basal production	Our results (Figure EV3B)
	→	IFI44L_LC_1	synthIFI44LmRNA · $\frac{\text{OccISREbs}^{\text{n\_IFI44L}}}{\text{kmIFI44L}^{\text{n\_IFI44L}}+\text{OccISREbs}^{\text{n\_IFI44L}}}$	IFI44LmRNA production with Hill kinetic and linear chain for time delay	Our results (Figure EV3B)
IFI44L_LC_1	→	IFI44L_LC_2	delayIFI44LmRNA · IFI44L_LC_1	IFI44L chain step 1	(MacDonald, 1976)
IFI44L_LC_2	→	IFI44L_LC_3	delayIFI44LmRNA · IFI44L_LC_2	IFI44L chain step 2	(MacDonald, 1976)
IFI44L_LC_3	→	IFI44LmRNA	delayIFI44LmRNA ·IFI44L_LC_3	IFI44L chain step 3	(MacDonald, 1976)
IFI44LmRNA	→		hlIFI44LmRNA ·IFI44LmRNA	IFI44LmRNA decay	Our results (Figure EV3B)

Appendix Table S1: Model reactions
Each row corresponds to a molecular reaction as indicated by the model structure (Fig. 2). Rates were derived from mass-action kinetics including Michaelis-Menten terms. Justifications are given in the right hand column based on published literature or own data.

Appendix Table S2

Measured Component	Computation by means of model states, scaling and offset parameters
**Protein**	
$\text{pSTAT1}_{\text{Nuc}}$	log(scale_pSTAT1_Nuc · (ISGF3n + 2 · pSTAT1dimn + pSTAT1pSTAT2n) + off_pSTAT1_Nuc)
$\text{pSTAT1}_{\text{Cyt}}$	log(scale_pSTAT1_Cyt · (ISGF3c + 2 · pSTAT1dimc + pSTAT1pSTAT2c) + off_pSTAT1_Cyt)
pSTAT1	log(scale_pSTAT1· (ISGF3c + ISGF3n + 2 · pSTAT1dimc + pSTAT1pSTAT2c + 2 · pSTAT1dimn + pSTAT1pSTAT2n) + off_pSTAT1)
$\text{pSTAT2}_{\text{Nuc}}$	log(scale_pSTAT2_Nuc · (ISGF3n + pSTAT1pSTAT2n) + off_pSTAT2_Nuc)
$\text{pSTAT2}_{\text{Cyt}}$	log(scale_pSTAT2_Cyt · (ISGF3c + pSTAT1pSTAT2c) + off_pSTAT2_Cyt)
pSTAT2	log(scale_pSTAT2 · (ISGF3c + ISGF3n + pSTAT1pSTAT2c + pSTAT1pSTAT2n) + off_pSTAT2)
IRF9_{Nuc}	log(scale_IRF9_Nuc · (ISGF3n + IRF9n) + off_IRF9_Nuc)

Measured Component	Computation by means of model states, scaling and offset parameters
$IRF9_{Cyt}$	log(scale_IRF9_Cyt · (ISGF3c + IRF9c) + off_IRF9_Cyt)
IRF9	log(scale_IRF9 · (ISGF3c + ISGF3n + IRF9c + IRF9n) + off_IRF9)
USP18 / $USP18_{Cyt}$	log(scale_USP18 · USP18 + off_USP18)
$tSTAT1_{Nuc}$	log(scale_tSTAT1_Nuc · (ISGF3n + STAT1n + 2 · pSTAT1dimn + pSTAT1pSTAT2n) + off_tSTAT1_Nuc)
$tSTAT1_{Cyt}$	log(scale_tSTAT1_Cyt · (ISGF3c + STAT1c + 2 · pSTAT1dimc + pSTAT1pSTAT2c) + off_tSTAT1_Cyt)
tSTAT1	log(scale_tSTAT1 · (ISGF3c + STAT1c + 2 · pSTAT1dimc + pSTAT1pSTAT2c + ISGF3n + STAT1n + 2 · pSTAT1dimn + pSTAT1pSTAT2n) + off_tSTAT1)
$tSTAT2_{Nuc}$	log(scale_tSTAT2_Nuc · (ISGF3n + STAT2n + pSTAT1pSTAT2n) + off_tSTAT2_Nuc)
$tSTAT2_{Cyt}$	log(scale_tSTAT2_Cyt · (ISGF3c + STAT2c + pSTAT1pSTAT2c) + off_tSTAT2_Cyt)
tSTAT2	log(scale_tSTAT2 · (ISGF3c + STAT2c + pSTAT1pSTAT2c + ISGF3n + STAT2n + pSTAT1pSTAT2n) + off_tSTAT2)
**mRNA**	
STAT1mRNA	log(STAT1mRNA)
STAT2mRNA	log(STAT2mRNA)
IRF9mRNA	log(IRF9mRNA)
IRF2mRNA	log(IRF2mRNA)
USP18mRNA	log(USP18mRNA)
SOCS1mRNA	log(SOCS1mRNA)
SOCS3mRNA	log(SOCS3mRNA)
**Molecules per cell**	
STAT1	ISGF3n + ISGF3c + STAT1n + STAT1c + 2 · pSTAT1dimc + 2 · pSTAT1dimn + pSTAT1pSTAT2c + pSTAT1pSTAT2n
STAT2	ISGF3n + ISGF3c + STAT2n + STAT2c + pSTAT1pSTAT2c + pSTAT1pSTAT2n
IRF9	ISGF3n + ISGF3c + IRF9c + IRF9n
USP18	USP18
**Protein and mRNA for model validation**	
SOCS3	log(SOCS3)
IRF1mRNA	log(IRF1mRNA)
SOCS3mRNA	log(SOCS3mRNA)
DDX58mRNA	log(DDX58mRNA)
HERC5mRNA	log(HERC5mRNA)
IFI44LmRNA	log(IFI44LmRNA)
GAS_EMSA	OccGASbs
IB_pSTAT1_IP_STAT2	pSTAT1pSTAT2c + pSTAT1pSTAT2n + ISGF3c + ISGF3n
IB_pSTAT1_IP_IRF9	ISGF3c + ISGF3n

Appendix Table S2: Observables of the model
Observables were computed with respect to model states as indicated. In many cases, the proteins appear in various components of the model but only the sum of the components was accessible by the experiment. If the measurement technique only provides values on an arbitrary scale, scaling and offset factors were introduced that were estimated from the experimental data.

Appendix Table S3

Name of parameter	Estimated value (log)	95%-confidence interval [log(lower bound)]	95%-confidence interval [log(upper bound)]
Rec	3.0	2.9	3.1
totSTAT1	0.29	0.18	0.40

Name of parameter	Estimated value (log)	95%-confidence interval [log(lower bound)]	95%-confidence interval [log(upper bound)]
totSTAT2	-1.1	-1.7	-0.7
totIRF9	0.73	0.44	0.99
synthUSP18	0.5	-0.2	1.3
synthUSP18mRNAbasal	-2.7	-2.8	-2.6
hlUSP18mRNA	-2.4	-2.5	-2.3
degUSP18	-0.4	-1	0.33
synthSTAT1mRNAbasal	-1.4	-1.5	-1.3
hlSTAT1mRNA	-1.4	-1.5	-1.4
synthSTAT2mRNAbasal	-1.6	-1.7	-1.5
hlSTAT2mRNA	-1.4	-1.5	-1.3
synthIRF9mRNAbasal	-1.0	-1.1	-0.94
hlIRF9mRNA	-0.87	-0.94	-0.8
synthIRF2mRNAbasal	-0.02	-0.17	0.15
delayIRF2	0.06	-0.09	0.22
synthSOCS1mRNAbasal	0.26	0.07	0.47
hlSOCS1mRNA	-0.57	-0.86	-0.28
IRF2	0.58	0.25	0.89
synthSOCS3mRNAbasal	0.23	0.05	0.44
hlSOCS3mRNA	0.15	-0.02	0.35
degSTAT1	-0.69	-1.24	0.24
delaySTAT1	-0.64	-0.87	-0.37
degSTAT2	-1.9	-2.1	-1.7
delaySTAT2	0.57	0.38	0.80
degIRF9	-1.6	-1.8	-1.4
delayIRF9	0.13	-0.1	0.37
delayUSP18	-0.4	-0.7	0.0
degSOCS1	-0.10	-0.43	0.38
delaySOCS1	-0.10	-0.43	0.38
BindIFN	-2.8	-7.2	-
kinhBySOCS	6.8	2.3	-
kinhByUSP18	-8.9	-9.6	-8.2
degRec	-11	-	-7.5
degRecBySOCS	-4.6	-4.8	-4.4
degaRecIFN	3.2	1.8	-
degaRecIFNBySOCS	-0.12	-	-
degByUSP18	-4.1	-	11
degBySOCSandUSP18	-3.4	-4.9	-
ComplFormSTAT1STAT2c	-13.5	-15	-
TrLocSTAT1STAT2c	-1.7	-1.8	-1.6
BindIRF9c	-4.7	-5.7	-3
decaySTAT1STAT2n	0.17	0.037	0.31
TrLocISGF3c	-0.15	-0.24	-0.04
decayISGF3n	1.2	1.1	1.4
ComplFormSTAT1dimc	-8.1	-9.7	-
TrLocSTAT1n	-0.03	-0.55	0.51
decaySTAT1dimn	2.4	1.9	2.7
TrLocSTAT2n	0.59	0.35	0.85
TrLocSTAT1dimc	1.5	1.1	1.9

Name of parameter	Estimated value (log)	95%-confidence interval [log(lower bound)]	95%-confidence interval [log(upper bound)]
hlOccGASbs+OccISREbs	-0.59	-0.68	-0.50
genOccGASbs+OccISREbsBySTAT1STAT2n	-8.1	-8.3	-7.9
hlOccGASbs	7.2	3.3	-
synthSTAT1mRNA	1.5	1.4	1.6
kmSTAT1	-0.02	-0.26	0.21
synthSTAT2mRNA	0.45	0.24	0.66
synthIRF9mRNA	2.92	2.86	2.99
kmIRF9	-2.1	-2.4	-1.9
synthIRF2mRNA	-0.39	-0.63	-0.13
synthUSP18mRNA	1.0	0.8	1.3
synthSOCS1mRNA	2.2	1.9	2.5
synthSOCS3mRNA	-4.4	-4.7	-4.1
degSOCS3	7.6	3.9	-
kmsynthSTAT1	1.7	1.5	1.9
scale_pSTAT1_Nuc	0.93	0.73	1.15
off_pSTAT1_Nuc	-2.2	-2.3	-2
scale_pSTAT1_Cyt	-0.94	-1.1	-0.74
off_pSTAT1_Cyt	-1.4	-1.5	-1.3
scale_pSTAT2_Nuc	0.75	0.55	0.97
off_pSTAT2_Nuc	-0.35	-0.45	-0.26
scale_pSTAT2_Cyt	-1	-1.2	-0.85
off_pSTAT2_Cyt	-0.20	-0.29	-0.13
scale_IRF9_Cyt	-2.4	-2.4	-2.3
scale_IRF9_Nuc	-0.81	-0.89	-0.73
scale_USP18	-9.3	-9.6	-9.0
scale_tSTAT1_Nuc	2.2	2.1	2.3
scale_tSTAT1_Cyt	-0.42	-0.54	-0.31
off_tSTAT1_Cyt	-0.14	-0.25	-0.05
scale_tSTAT2_Nuc	1.5	1.4	1.5
scale_tSTAT2_Cyt	-0.67	-0.73	-0.6
off_tSTAT2_Cyt	-1.4	-1.6	-1.2
synthUSP18mRNAbasal_OE	2.3	2.1	2.4
synthUSP18_inh	0.64	0.52	0.75
synthUSP18mRNAbasal_inh	0.93	0.75	1.1
synthUSP18mRNA_inh	2.8	2.7	2.9

Appendix Table S3: Estimated Huh7.5 parameters

Parameter values of the global optimum for the Huh7.5 core model and profile-likelihood based confidence intervals (compare Figure S12) are shown on logarithmic scale.

Appendix Table S4

Model parameter	Parameter and steady-state transformations (functions of estimated parameters)	Value
**Initial values**		
IFN	0	0
Rec	exp(Rec)·100	1989
aRecIFN	0	0
pSTAT1pSTAT2c	0	0
pSTAT1pSTAT2n	0	0

Model parameter	Parameter and steady-state transformations (functions of estimated parameters)	Value
ISGF3c	0	0
ISGF3n	0	0
STAT1c	400000·exp(totSTAT1)/(1+(1/10))	486300
STAT1n	400000·exp(totSTAT1)·(1/10)/(1+(1/10))	48630
STAT2c	25000·(1+exp(totSTAT2))/(1+(1/10))	30390
STAT2n	25000·(1+exp(totSTAT2))·(1/10)/(1+(1/10))	3039
pSTAT1dimc	0	0
pSTAT1dimn	0	0
IRF9c	500·(1+exp(totIRF9))/(1+(1/10))	1400
IRF9n	500·(1+exp(totIRF9))·(1/10)/(1+(1/10))	140
USP18	exp(synthUSP18)·1000·exp(synthUSP18mRNAbasal)/ exp(hlUSP18mRNA)/exp(degUSP18)	1746
OccGASbs+OccISREbs	0	0
OccGASbs	0	0
STAT1mRNA	exp(synthSTAT1mRNAbasal)/exp(hlSTAT1mRNA)	1.012
STAT2mRNA	exp(synthSTAT2mRNAbasal)/exp(hlSTAT2mRNA)	0.8458
IRF9mRNA	exp(synthIRF9mRNAbasal)/exp(hlIRF9mRNA)	0.8606
IRF2mRNA	exp(synthIRF2mRNAbasal)/exp(delayIRF2)	0.9258
USP18mRNA	exp(synthUSP18mRNAbasal)/exp(hlUSP18mRNA)	0.7077
SOCS1mRNA	exp(synthSOCS1mRNAbasal)/(exp(hlSOCS1mRNA)·(1+exp(IRF2)))	0.8286
SOCS3mRNA	exp(synthSOCS3mRNAbasal)/exp(hlSOCS3mRNA)	1.081
IRF2	exp(IRF2)	1.781
SOCS1	(exp(synthSOCS1mRNAbasal)/(exp(hlSOCS1mRNA)·(1+exp(IRF2))))	0.8286
SOCS3	(exp(synthSOCS3mRNAbasal)/exp(hlSOCS3mRNA))	1.081
STAT1_LC_1	400000·exp(totSTAT1)/(1+(1/10))·exp(degSTAT1)/exp(delaySTAT1)	460600
STAT1_LC_2	400000·exp(totSTAT1)/(1+(1/10))·exp(degSTAT1)/exp(delaySTAT1)	460600
STAT1_LC_3	400000·exp(totSTAT1)/(1+(1/10))·exp(degSTAT1)/exp(delaySTAT1)	460600
STAT2_LC_1	25000·(1+exp(totSTAT2))/(1+(1/10))·exp(degSTAT2)/exp(delaySTAT2)	2520
STAT2_LC_2	25000·(1+exp(totSTAT2))/(1+(1/10))·exp(degSTAT2)/exp(delaySTAT2)	2520
STAT2_LC_3	25000·(1+exp(totSTAT2))/(1+(1/10))·exp(degSTAT2)/exp(delaySTAT2)	2520
STAT2_LC_4	25000·(1+exp(totSTAT2))/(1+(1/10))·exp(degSTAT2)/exp(delaySTAT2)	2520
STAT2_LC_5	25000·(1+exp(totSTAT2))/(1+(1/10))·exp(degSTAT2)/exp(delaySTAT2)	2520
IRF9_LC_1	500·(1+exp(totIRF9))/(1+(1/10))·exp(degIRF9)/exp(delayIRF9)	249.7
IRF9_LC_2	500·(1+exp(totIRF9))/(1+(1/10))·exp(degIRF9)/exp(delayIRF9)	249.7
USP18_LC_1	exp(synthUSP18)·1000·exp(synthUSP18mRNAbasal)/ exp(hlUSP18mRNA)/exp(delayUSP18)	1745
USP18_LC_2	exp(synthUSP18)·1000·exp(synthUSP18mRNAbasal)/ exp(hlUSP18mRNA)/exp(delayUSP18)	1745
SOCS1_LC_1	exp(degSOCS1)·exp(synthSOCS1mRNAbasal)/ (exp(hlSOCS1mRNA)·(1+exp(IRF2)))/exp(delaySOCS1)	0.8286
**Dynamic parameters**		
BindIFN	exp(BindIFN)	0.0593
kinhBySOCS	exp(kinhBySOCS)	889.4
kinhByUSP18	exp(kinhByUSP18)	0.0001364
prodRec	exp(Rec)·100·(exp(degRec)+exp(degRecBySOCS)· exp(synthSOCS1mRNAbasal)/(exp(hlSOCS1mRNA)·(1+exp(IRF2))))	16.98
degRec	exp(degRec)	2.06e-05
degRecBySOCS	exp(degRecBySOCS)	0.01028
degaRecIFN	exp(degaRecIFN)	19840

Model parameter	Parameter and steady-state transformations (functions of estimated parameters)	Value
degaRecIFNBySOCS	exp(degaRecIFNBySOCS)	0.8837
degByUSP18	exp(degByUSP18)	0.01644
degBySOCSandUSP18	exp(degBySOCSandUSP18)	27.82
ComplFormSTAT1STAT2c	exp(ComplFormSTAT1STAT2c)	0.001112
TrLocSTAT1STAT2c	exp(TrLocSTAT1STAT2c)	0.1855
BindIRF9c	exp(BindIRF9c)	0.008975
decaySTAT1STAT2n	exp(decaySTAT1STAT2n)	1.187
TrLocISGF3c	exp(TrLocISGF3c)	0.8636
decayISGF3n	exp(decayISGF3n)	3.407
ComplFormSTAT1dimc	exp(ComplFormSTAT1dimc)/(25000·(1+exp(totSTAT2)))	7.12e-06
degSTAT1	exp(degSTAT1)	0.4996
TrLocSTAT1n	exp(TrLocSTAT1n)	0.9702
TrLocSTAT1c	exp(TrLocSTAT1n)·(1/10)	0.09702
delaySTAT1	exp(delaySTAT1)	0.5275
decaySTAT1dimn	exp(decaySTAT1dimn)	10.53
degSTAT2	exp(degSTAT2)	0.1468
TrLocSTAT2n	exp(TrLocSTAT2n)	1.81
TrLocSTAT2c	exp(TrLocSTAT2n)·(1/10)	0.181
delaySTAT2	exp(delaySTAT2)	1.77
TrLocSTAT1dimc	exp(TrLocSTAT1dimc)	4.296
TrLocIRF9n	2980.96	2981
TrLocIRF9c	(2980.96)·(1/10)	298.1
degIRF9	exp(degIRF9)	0.2028
delayIRF9	exp(delayIRF9)	1.137
degUSP18	exp(degUSP18)	0.6719
delayUSP18	exp(delayUSP18)	0.6722
genOccGASbs+ OccISREbsByISGF3n	exp(hlOccGASbs+OccISREbs)/(500·(1+exp(totIRF9)))	0.0003583
genOccGASbs+ OccISREbsBySTAT1STAT2n	exp(genOccGASbs+OccISREbsBySTAT1STAT2n)	0.0002972
hlOccGASbs+OccISREbs	exp(OccGASbs+OccISREbs)	0.5519
genOccGASbsBySTAT1dimn	exp(hlOccGASbs)	1395
hlOccGASbs	exp(hlOccGASbs)	1395
synthSTAT1mRNAbasal	exp(synthSTAT1mRNAbasal)	0.2395
synthSTAT1mRNA	exp(synthSTAT1mRNA)	4.375
kmSTAT1	exp(kmSTAT1)	0.9761
hlSTAT1mRNA	exp(hlSTAT1mRNA)	0.2367
synthSTAT2mRNAbasal	exp(synthSTAT2mRNAbasal)	0.2069
synthSTAT2mRNA	exp(synthSTAT2mRNA)	1.561
hlSTAT2mRNA	exp(hlSTAT2mRNA)	0.2446
synthIRF9mRNAbasal	exp(synthIRF9mRNAbasal)	0.3605
synthIRF9mRNA	exp(synthIRF9mRNA)	18.62
kmIRF9	exp(kmIRF9)	0.121
hlIRF9mRNA	exp(hlIRF9mRNA)	0.4189
synthIRF2mRNAbasal	exp(synthIRF2mRNAbasal)	0.9833
synthIRF2mRNA	exp(synthIRF2mRNA)	0.6775
delayIRF2	exp(delayIRF2)	1.062
synthUSP18mRNAbasal	exp(synthUSP18mRNAbasal)	0.06442
synthUSP18mRNA	exp(synthUSP18mRNA)	2.784

Model parameter	Parameter and steady-state transformations (functions of estimated parameters)	Value
hlUSP18mRNA	exp(hlUSP18mRNA)	0.09103
synthSOCS1mRNAbasal	exp(synthSOCS1mRNAbasal)	1.301
synthSOCS1mRNA	exp(synthSOCS1mRNA)	8.848
hlSOCS1mRNA	exp(hlSOCS1mRNA)	0.5644
synthSOCS3mRNAbasal	exp(synthSOCS3mRNAbasal)	1.257
synthSOCS3mRNA	exp(synthSOCS3mRNA)	0.01205
hlSOCS3mRNA	exp(hlSOCS3mRNA)	1.163
degIRF2	(exp(synthIRF2mRNAbasal)/exp(delayIRF2))· exp(delayIRF2)/exp(IRF2)	0.552
degSOCS1	exp(degSOCS1)	0.9029
delaySOCS1	exp(delaySOCS1)	0.9029
synthSOCS3	exp(degSOCS3)	1958
degSOCS3	exp(degSOCS3)	1958
synthSTAT1	400000·exp(totSTAT1)/(1+(1/10))·exp(degSTAT1)/ (exp(synthSTAT1mRNAbasal)/exp(hlSTAT1mRNA))· (exp(kmsynthSTAT1)+exp(synthSTAT1mRNAbasal)/exp(hlSTAT1mRNA))	1533000
kmsynthSTAT1	exp(kmsynthSTAT1)	5.373
synthSTAT2	25000·(1+exp(totSTAT2))/(1+(1/10))·exp(degSTAT2)/ (exp(synthSTAT2mRNAbasal)/exp(hlSTAT2mRNA))	5274
synthIRF9	500·(1+exp(totIRF9))/(1+(1/10))·exp(degIRF9)/ (exp(synthIRF9mRNAbasal)/exp(hlIRF9mRNA))	329.9
synthUSP18	exp(synthUSP18)·1000	1658
synthSOCS1	exp(degSOCS1)	0.9029
**Scaling and offset parameters**		
scale_pSTAT1_Nuc	exp(scale_pSTAT1_Nuc)/(500·(1+exp(totIRF9)))	0.001652
off_pSTAT1_Nuc	exp(off_pSTAT1_Nuc)	0.1137
scale_pSTAT1_Cyt	exp(scale_pSTAT1_Cyt)/(500·(1+exp(totIRF9)))	0.0002546
off_pSTAT1_Cyt	exp(off_pSTAT1_Cyt)	0.248
scale_pSTAT2_Nuc	exp(scale_pSTAT2_Nuc)/(500·(1+exp(totIRF9)))	0.001381
off_pSTAT2_Nuc	exp(off_pSTAT2_Nuc)	0.7026
scale_pSTAT2_Cyt	exp(scale_pSTAT2_Cyt)/(500·(1+exp(totIRF9)))	0.0002293
off_pSTAT2_Cyt	exp(off_pSTAT2_Cyt)	0.8152
scale_IRF9_Cyt	exp(scale_IRF9_Cyt)/(500·(1+exp(totIRF9)))	6.108e-05
off_IRF9_Cyt	0	0
scale_IRF9_Nuc	exp(scale_IRF9_Nuc)/(500·(1+exp(totIRF9)))	0.0002884
off_IRF9_Nuc	0	0
scale_USP18	exp(scale_USP18)	9.17e-05
off_USP18	0	0
scale_tSTAT1_Nuc	exp(scale_tSTAT1_Nuc)/(400000·exp(totSTAT1))	1.701e-05
off_tSTAT1_Nuc	0	0
scale_tSTAT1_Cyt	exp(scale_tSTAT1_Cyt)/(400000·exp(totSTAT1))	1.224e-06
off_tSTAT1_Cyt	exp(off_tSTAT1_Cyt)	0.8691
scale_tSTAT2_Nuc	exp(scale_tSTAT2_Nuc)/(25000·(1+exp(totSTAT2)))	0.0001279
off_tSTAT2_Nuc	0	0
scale_tSTAT2_Cyt	exp(scale_tSTAT2_Cyt)/(25000·(1+exp(totSTAT2)))	1.536e-05
off_tSTAT2_Cyt	exp(off_tSTAT2_Cyt)	0.2489

Model parameter	Parameter and steady-state transformations (functions of estimated parameters)	Value

Appendix Table S4: Model parameters for Huh7.5
Model parameters were grouped into initial values, dynamic parameters as well as scaling and offset parameters introduced via the observables. Initial values correspond to model states at beginning of the integration (time point 0h). Dynamic parameters correspond to molecular reactions given in Table S1. Scaling and offset parameters were introduced by means of observable functions (Table S2). Steady-state and parameter transformations indicate how model parameters were computed from the estimated parameters.

Appendix Table S5

Name of parameter	Value	Name of parameter	Value
totSTAT1	0.5208	scale_IRF9_Cyt	-2.239
totSTAT2	-4.859	off_IRF9_Cyt	-3.228
totIRF9	2.076	scale_IRF9_Nuc	-0.4322
synthUSP18	2.481	off_IRF9_Nuc	-1.627
ratio_synthSTAT1mRNAbasal	-1.777	scale_USP18	-10.52
ratio_ComplFormSTAT1STAT2c	0.9606	off_USP18	-1.918
scale_pSTAT1_Nuc	1.511	scale_tSTAT1_Nuc	0.2964
off_pSTAT1_Nuc	-1.677	off_tSTAT1_Nuc	259
scale_pSTAT1_Cyt	-0.2207	scale_tSTAT1_Cyt	-1.992
off_pSTAT1_Cyt	-1.01	off_tSTAT1_Cyt	0.5588
scale_pSTAT2_Nuc	1.204	scale_tSTAT2_Nuc	1.576
off_pSTAT2_Nuc	0.3613	off_tSTAT2_Nuc	-1.009
scale_pSTAT2_Cyt	-0.96	scale_tSTAT2_Cyt	-0.2864
off_pSTAT2_Cyt	0.6448	off_tSTAT2_Cyt	0.06068

Appendix Table S5: Estimated HepG2-hNTCP parameters
Only parameters different from the Huh7.5 parameters (Table S3) were shown. Parameters are shown on logarithmic scale.

Appendix Table S6

Name of parameter	Value	Name of parameter	Value
BindIFN	0.1044	synthSTAT1	9911000
ComplFormSTAT1STAT2c	0.002907	synthSTAT2	3975
synthSTAT1mRNAbasal	0.0405	synthIRF9	960.9
STAT1c	2290	synthUSP18	11950
STAT1n	4078	scale_pSTAT1_Nuc	0.00101
STAT2c	407.8	off_pSTAT1_Nuc	0.1869
STAT2n	12590	scale_pSTAT1_Cyt	0.0001788
IRF9c	0.1711	off_pSTAT1_Cyt	0.3641
IRF9n	0.8458	scale_pSTAT2_Nuc	0.0007431
USP18	0.8606	off_pSTAT2_Nuc	1.435
STAT1mRNA	0.7077	scale_pSTAT2_Cyt	8,535e-05
STAT2mRNA	579800	off_pSTAT2_Cyt	1.906
IRF9mRNA	579800	scale_IRF9_Cyt	2,376e-05
USP18mRNA	579800	off_IRF9_Cyt	0.03965
STAT1_LC_1	1900	scale_IRF9_Nuc	0.0001447
STAT1_LC_2	1900	off_IRF9_Nuc	0.1966

Name of parameter	Value	Name of parameter	Value
STAT1_LC_3	1900	scale_USP18	2.699e-05
STAT2_LC_1	1900	off_USP18	0.1468
STAT2_LC_2	1900	scale_tSTAT1_Nuc	1.997e-06
STAT2_LC_3	727.1	off_tSTAT1_Nuc	1.296
STAT2_LC_4	727.1	scale_tSTAT1_Cyt	2,026e-07
STAT2_LC_5	12580	off_tSTAT1_Cyt	1.749
IRF9_LC_1	12580	scale_tSTAT2_Nuc	0.000192
IRF9_LC_2	0.8286	off_tSTAT2_Nuc	0.3647
USP18_LC_1	0.1044	scale_tSTAT2_Cyt	2,981e-05
USP18_LC_2	0.002907	off_tSTAT2_Cyt	1.063
SOCS1_LC_1	0.0405		

Appendix Table S6: Model parameters for HepG2-hNTCP
Only parameters different from the Huh7.5 parameters (Table S4) were shown.

Appendix Table S7

Patient	Name of parameter	Value	Patient	Name of parameter	Value
all	ratio_Rec	1.566	3	scale_tSTAT2_Cyt_pat3	0.1424
all	ratio_synthSTAT2mRNAbasal	-0.1346	3	off_tSTAT2_Cyt_pat3	-1.848
all	ratio_hlSOCS1mRNA	1.756	4	totSTAT1_pat4	1.022
all	ratio_decayISGF3n	-1.085	4	totSTAT2_pat4	1.366
all	ratio_synthSTAT2mRNA	0.1346	4	totIRF9_pat4	6.559
1	totSTAT1_pat1	-0.826	4	synthUSP18_pat4	3.117
1	totSTAT2_pat1	0.291	4	scale_pSTAT1_Cyt_pat4	4.375
1	totIRF9_pat1	5.017	4	off_pSTAT1_Cyt_pat4	0.3007
1	synthUSP18_pat1	2.885	4	scale_pSTAT2_Cyt_pat4	2.229
1	scale_pSTAT1_Cyt_pat1	5.902	4	off_pSTAT2_Cyt_pat4	0.06427
1	off_pSTAT1_Cyt_pat1	0.5444	4	scale_IRF9_Cyt_pat4	-2.928
1	scale_pSTAT2_Cyt_pat1	2.747	4	off_IRF9_Cyt_pat4	-0.2581
1	off_pSTAT2_Cyt_pat1	0.7861	4	scale_USP18_pat4	-11.54
1	scale_IRF9_Cyt_pat1	-1.191	4	off_USP18_pat4	-1.09
1	off_IRF9_Cyt_pat1	0.4407	4	scale_tSTAT1_Cyt_pat4	-0.4432
1	scale_USP18_pat1	-10.15	4	off_tSTAT1_Cyt_pat4	-1.21
1	off_USP18_pat1	-4.632	4	scale_tSTAT2_Cyt_pat4	-1.493
1	scale_tSTAT1_Cyt_pat1	0.3616	4	off_tSTAT2_Cyt_pat4	0.06455
1	off_tSTAT1_Cyt_pat1	-4.356	5	totSTAT1_pat5	0.9405
1	scale_tSTAT2_Cyt_pat1	-0.3356	5	totSTAT2_pat5	2.265
1	off_tSTAT2_Cyt_pat1	-6.226	5	totIRF9_pat5	5.399
2	totSTAT1_pat2	-0.8074	5	synthUSP18_pat5	3.336
2	totSTAT2_pat2	-0.1972	5	scale_pSTAT1_Cyt_pat5	2.936
2	totIRF9_pat2	4.256	5	off_pSTAT1_Cyt_pat5	0.1039
2	synthUSP18_pat2	4.036	5	scale_pSTAT2_Cyt_pat5	0.8225
2	scale_pSTAT1_Cyt_pat2	3.725	5	off_pSTAT2_Cyt_pat5	0.7362
2	off_pSTAT1_Cyt_pat2	0.5595	5	scale_IRF9_Cyt_pat5	-2.736
2	scale_pSTAT2_Cyt_pat2	3.837	5	off_IRF9_Cyt_pat5	0.03525
2	off_pSTAT2_Cyt_pat2	1.184	5	scale_USP18_pat5	-13.3
2	scale_IRF9_Cyt_pat2	-2.461	5	off_USP18_pat5	-0.7422
2	off_IRF9_Cyt_pat2	-0.1382	5	scale_tSTAT1_Cyt_pat5	-0.6165
2	scale_USP18_pat2	-10.75	5	off_tSTAT1_Cyt_pat5	-0.7407

Patient	Name of parameter	Value	Patient	Name of parameter	Value
2	off_USP18_pat2	-1.031	5	scale_tSTAT2_Cyt_pat5	-2.774
2	scale_tSTAT1_Cyt_pat2	-0.8868	5	off_tSTAT2_Cyt_pat5	-0.1237
2	off_tSTAT1_Cyt_pat2	-0.1189	6	totSTAT1_pat6	0.75
2	scale_tSTAT2_Cyt_pat2	-0.5563	6	totSTAT2_pat6	1.007
2	off_tSTAT2_Cyt_pat2	-0.05393	6	totIRF9_pat6	3.817
3	totSTAT1_pat3	-0.5841	6	synthUSP18_pat6	3.183
3	totSTAT2_pat3	-6.104	6	scale_pSTAT1_Cyt_pat6	2.202
3	totIRF9_pat3	4.264	6	off_pSTAT1_Cyt_pat6	0.5528
3	synthUSP18_pat3	1.564	6	scale_pSTAT2_Cyt_pat6	-0.7454
3	scale_pSTAT1_Cyt_pat3	3.72	6	off_pSTAT2_Cyt_pat6	0.2017
3	off_pSTAT1_Cyt_pat3	-0.6271	6	scale_IRF9_Cyt_pat6	-2.498
3	scale_pSTAT2_Cyt_pat3	4.745	6	off_IRF9_Cyt_pat6	-0.4419
3	off_pSTAT2_Cyt_pat3	0.2139	6	scale_USP18_pat6	-12.07
3	scale_IRF9_Cyt_pat3	-2.969	6	off_USP18_pat6	-0.1461
3	off_IRF9_Cyt_pat3	-0.4246	6	scale_tSTAT1_Cyt_pat6	-0.5982
3	scale_USP18_pat3	-9.448	6	off_tSTAT1_Cyt_pat6	0.2037
3	off_USP18_pat3	-0.4344	6	scale_tSTAT2_Cyt_pat6	-1.782
3	scale_tSTAT1_Cyt_pat3	-0.09469	6	off_tSTAT2_Cyt_pat6	0.424
3	off_tSTAT1_Cyt_pat3	-5.667			

Appendix Table S7: Estimated primary human hepatocyte parameters
For each patient, only parameters different from the Huh7.5 parameters (Table S3) were shown. Parameters were shown on logarithmic scale.

Appendix Table S8

Name of parameter	Estimated value (log)	95%-confidence interval [log(lower bound)]	95%-confidence interval [log(upper bound)]	Value
**Huh7.5**				
synthDDX58mRNAbasal	1.53	1.23	1.92	4.63
delayDDX58mRNA	1.45	1.34	1.57	4.27
hlDDX58mRNA	1.45	1.16	1.83	4.27
synthHERC5mRNAbasal	-0.146	-0.232	-0.0545	0.864
delayHERC5mRNA	2.37	2.23	2.52	10.7
hlHERC5mRNA	-0.164	-0.25	-0.0721	0.849
synthIFI44LmRNAbasal	0.149	-0.178	0.501	1.16
delayIFI44LmRNA	0.496	0.396	0.601	1.64
hlIFI44LmRNA	0.5	0.214	0.82	1.64
synthIRF1mRNAbasal	0.377	0.231	0.523	1.46
delayIRF1mRNA	2.09	1.97	2.22	8.09
hlIRF1mRNA	0.221	0.139	0.314	1.25
synthSOCS3mRNAbasal	0.0265	-0.124	0.195	1.030
delaySOCS3mRNA	2.71	2.41	3.15	15.0
hlSOCS3mRNA	-0.0282	-0.154	0.128	0.972
synthDDX58mRNA	5.98	5.64	6.4	396
n_DDX58	0.371	0.31	0.433	1.45
kmDDX58	0.324	0.124	0.572	1.38
synthHERC5mRNA	3.27	3.2	3.35	26.3
n_HERC5	1.02	0.959	1.08	2.78

Name of parameter	Estimated value (log)	95%-confidence interval [log(lower bound)]	95%-confidence interval [log(upper bound)]	Value
kmHERC5	-0.537	-0.592	-0.474	0.585
synthIFI44LmRNA	7.37	7.09	7.7	1594
n_IFI44L	1.47	1.42	1.52	4.35
kmIFI44L	-0.578	-0.605	-0.548	0.561
synthIRF1mRNA	4.11	3.97	4.28	61.2
n_IRF1	0.258	0.193	0.331	1.29
kmIRF1	5.49	5.3	5.68	241
synthSOCS3mRNA	4.85	3.19	NA	127
n_SOCS3	0.0221	-0.112	0.28	1.02
kmSOCS3	9.63	7.44	NA	1518
**HepG2-hNTCP**				
synthDDX58mRNAbasal	-1.74	n.c.	n.c.	0.18
delayDDX58mRNA	9.15	n.c.	n.c.	9.42
hlDDX58mRNA	-1.59	n.c.	n.c.	2.04
synthHERC5mRNAbasal	-3.65	n.c.	n.c.	2.61
delayHERC5mRNA	3.81	n.c.	n.c.	4.53
hlHERC5mRNA	-3.07	n.c.	n.c.	4.65
synthIFI44LmRNAbasal	3.29	n.c.	n.c.	3.74
delayIFI44LmRNA	-6.32	n.c.	n.c.	2.32
hlIFI44LmRNA	-2.18	n.c.	n.c.	0.08
synthSOCS3mRNAbasal	-2.00	n.c.	n.c.	1.030
delaySOCS3mRNA	-6.32	n.c.	n.c.	0.002
hlSOCS3mRNA	-2.18	n.c.	n.c.	0.11
synthDDX58mRNA	3.78	n.c.	n.c.	43.7
n_DDX58	0.69	n.c.	n.c.	2.00
kmDDX58	0.19	n.c.	n.c.	1.21
synthHERC5mRNA	4.89	n.c.	n.c.	134
n_HERC5	2.05	n.c.	n.c.	7.79
kmHERC5	-0.50	n.c.	n.c.	0.60
synthIFI44LmRNA	5.00	n.c.	n.c.	148
n_IFI44L	2.31	n.c.	n.c.	10.0
kmIFI44L	-0.68	n.c.	n.c.	0.50
synthSOCS3mRNA	-1.45	n.c.	n.c.	0.23
n_SOCS3	0	n.c.	n.c.	1.00
kmSOCS3	0.07	n.c.	n.c.	1.07

Appendix Table S8: Model parameters for validation model
Parameters used for fitting the validation data were shown. Parameters were shown on log-scale with profile-likelihood based confidence intervals. Confidence intervals for the L_1 regularization-based HepG2-hNTCP validation model were not computed (n.c.).

References

Banninger, G. and Reich, N. C. (2004). STAT2 nuclear trafficking. J Biol Chem *279*, 39199–206.

Bhattacharya, S., HuangFu, W. C., Liu, J., Veeranki, S., Baker, D. P., Koumenis, C., Diehl, J. A. and Fuchs, S. Y. (2010). Inducible priming phosphorylation promotes ligand-independent degradation of the IFNAR1 chain of type I interferon receptor. J Biol Chem *285*, 2318–25.

Chen, X. P., Losman, J. A. and Rothman, P. (2000). SOCS proteins, regulators of intracellular signaling. Immunity *13*, 287–90.

Decker, T., Lew, D. J. and Darnell, J. E., J. (1991). Two distinct alpha-interferon-dependent signal transduction pathways may contribute to activation of transcription of the guanylate-binding protein gene. Mol Cell Biol *11*, 5147–53.

Ehlting, C., Lai, W. S., Schaper, F., Brenndorfer, E. D., Matthes, R. J., Heinrich, P. C., Ludwig, S., Blackshear, P. J., Gaestel, M., Haussinger, D. and Bode, J. G. (2007). Regulation of suppressor of cytokine signaling 3 (SOCS3) mRNA stability by TNF-alpha involves activation of the MKK6/p38MAPK/MK2 cascade. J Immunol *178*, 2813–26.

Harada, H., Fujita, T., Miyamoto, M., Kimura, Y., Maruyama, M., Furia, A., Miyata, T. and Taniguchi, T. (1989). Structurally similar but functionally distinct factors, IRF-1 and IRF-2, bind to the same regulatory elements of IFN and IFN-inducible genes. Cell *58*, 729–39.

Harada, H., Matsumoto, M., Sato, M., Kashiwazaki, Y., Kimura, T., Kitagawa, M., Yokochi, T., Tan, R. S., Takasugi, T., Kadokawa, Y., Schindler, C., Schreiber, R. D., Noguchi, S. and Taniguchi, T. (1996). Regulation of IFN-alpha/beta genes: evidence for a dual function of the transcription factor complex ISGF3 in the production and action of IFN-alpha/beta. Genes Cells *1*, 995–1005.

Ilangumaran, S., Ramanathan, S. and Rottapel, R. (2004). Regulation of the immune system by SOCS family adaptor proteins. Semin Immunol *16*, 351–65.

Kumar, K. G., Tang, W., Ravindranath, A. K., Clark, W. A., Croze, E. and Fuchs, S. Y. (2003). SCF(HOS) ubiquitin ligase mediates the ligand-induced down-regulation of the interferon-alpha receptor. EMBO J *22*, 5480–90.

Lau, J. F., Parisien, J. P. and Horvath, C. M. (2000). Interferon regulatory factor subcellular localization is determined by a bipartite nuclear localization signal in the DNA-binding domain and interaction with cytoplasmic retention factors. Proc Natl Acad Sci U S A *97*, 7278–83.

Le Novere, N. (2015). Quantitative and logic modelling of molecular and gene networks. Nat Rev Genet *16*, 146–58.

Lehtonen, A., Matikainen, S. and Julkunen, I. (1997). Interferons up-regulate STAT1, STAT2, and IRF family transcription factor gene expression in human peripheral blood mononuclear cells and macrophages. J Immunol *159*, 794–803.

Li, X., Leung, S., Qureshi, S., Darnell, J. E., J. and Stark, G. R. (1996). Formation of STAT1-STAT2 heterodimers and their role in the activation of IRF-1 gene transcription by interferon-alpha. J Biol Chem *271*, 5790–4.

MacDonald, N. (1976). Time delay in simple chemostat models. Biotechnol Bioeng *18*, 805–12.

Malakhova, O. A., Kim, K. I., Luo, J. K., Zou, W., Kumar, K. G., Fuchs, S. Y., Shuai, K. and Zhang, D. E. (2006). UBP43 is a novel regulator of interferon signaling independent of its ISG15 isopeptidase activity. EMBO J *25*, 2358–67.

Mertens, C., Zhong, M., Krishnaraj, R., Zou, W., Chen, X. and Darnell, J. E., J. (2006). Dephosphorylation of phosphotyrosine on STAT1 dimers requires extensive spatial reorientation of the monomers facilitated by the N-terminal domain. Genes Dev *20*, 3372–81.

Meyer, T., Begitt, A., Lodige, I., van Rossum, M. and Vinkemeier, U. (2002). Constitutive and IFN-gamma-induced nuclear import of STAT1 proceed through independent pathways. EMBO J *21*, 344–54.

Novick, D., Cohen, B. and Rubinstein, M. (1994). The human interferon alpha/beta receptor: characterization and molecular cloning. Cell *77*, 391–400.

Paul, A., Tang, T. H. and Ng, S. K. (2018). Interferon Regulatory Factor 9 Structure and Regulation. Front Immunol *9*, 1831.

Piganis, R. A., De Weerd, N. A., Gould, J. A., Schindler, C. W., Mansell, A., Nicholson, S. E. and Hertzog, P. J. (2011). Suppressor of cytokine signaling (SOCS) 1 inhibits type I interferon (IFN) signaling via the interferon alpha receptor (IFNAR1)-associated tyrosine kinase Tyk2. J Biol Chem *286*, 33811–8.

Platanias, L. C. (2005). Mechanisms of type-I- and type-II-interferon-mediated signalling. Nat Rev Immunol *5*, 375–86.

Raue, A., Kreutz, C., Maiwald, T., Bachmann, J., Schilling, M., Klingmuller, U. and Timmer, J. (2009). Structural and practical identifiability analysis of partially observed dynamical models by exploiting the profile likelihood. Bioinformatics *25*, 1923–9.

Raue, A., Schilling, M., Bachmann, J., Matteson, A., Schelker, M., Kaschek, D., Hug, S., Kreutz, C., Harms, B. D., Theis, F. J., Klingmuller, U. and Timmer, J. (2013). Lessons learned from quantitative dynamical modeling in systems biology. PLoS One *8*, e74335.

Sarasin-Filipowicz, M., Wang, X., Yan, M., Duong, F. H., Poli, V., Hilton, D. J., Zhang, D. E. and Heim, M. H. (2009). Alpha interferon induces long-lasting refractoriness of JAK-STAT signaling in the mouse liver through induction of USP18/UBP43. Mol Cell Biol *29*, 4841–51.

Schindler, C., Shuai, K., Prezioso, V. R. and Darnell, J. E., J. (1992). Interferon-dependent tyrosine phosphorylation of a latent cytoplasmic transcription factor. Science *257*, 809–13.

Shuai, K. and Liu, B. (2003). Regulation of JAK-STAT signalling in the immune system. Nat Rev Immunol *3*, 900–11.

Siewert, E., Muller-Esterl, W., Starr, R., Heinrich, P. C. and Schaper, F. (1999). Different protein turnover of interleukin-6-type cytokine signalling components. Eur J Biochem *265*, 251–7.

Song, M. M. and Shuai, K. (1998). The suppressor of cytokine signaling (SOCS) 1 and SOCS3 but not SOCS2 proteins inhibit interferon-mediated antiviral and antiproliferative activities. J Biol Chem *273*, 35056–62.

Taniguchi, T., Ogasawara, K., Takaoka, A. and Tanaka, N. (2001). IRF family of transcription factors as regulators of host defense. Annu Rev Immunol *19*, 623–55.

Zhang, X., Bogunovic, D., Payelle-Brogard, B., Francois-Newton, V., Speer, S. D., Yuan, C., Volpi, S., Li, Z., Sanal, O., Mansouri, D., Tezcan, I., Rice, G. I., Chen, C., Mansouri, N., Mahdaviani, S. A., Itan, Y., Boisson, B., Okada, S., Zeng, L., Wang, X., Jiang, H., Liu, W., Han, T., Liu, D., Ma, T., Wang, B., Liu, M., Liu, J. Y., Wang, Q. K., Yalnizoglu, D., Radoshevich, L., Uze, G., Gros, P., Rozenberg, F., Zhang, S. Y., Jouanguy, E., Bustamante, J., Garcia-Sastre, A., Abel, L., Lebon, P., Notarangelo, L. D., Crow, Y. J., Boisson-Dupuis, S., Casanova, J. L. and Pellegrini, S. (2015). Human intracellular ISG15 prevents interferon-alpha/beta over-amplification and auto-inflammation. Nature *517*, 89–93.
