# Supplementary material for: Disentangling molecular mechanisms regulating sensitization of interferon alpha signal transduction
Source: Mol Syst Biol. 2020 Jul 21;16(7):e8955. doi: 10.15252/msb.20198955 (PMC7373899; doi:10.15252/msb.20198955)
Supplement: Supplementary file 2 — Expanded View Figures PDF [file MSB-16-e8955-s002.pdf]

Expanded View Figures

Figure EV1. Time- and dose-resolved quantitative data for model calibration.

A, B Model calibration with time-resolved (A) and IFNα dose-resolved (B) signal transduction. Huh7.5 cells were growth factor-depleted and stimulated with different doses of IFNα. Cytoplasmic and nuclear lysates were subjected to quantitative immunoblotting and protein signals detected with chemiluminescence using a CCD camera-based device. Data were normalized to reference proteins Calnexin or HDAC1 and represented by filled circles with errors representing 1σ confidence intervals estimated from biological replicates using a combined scaling and error model. Model trajectories are represented by solid lines. Yellow line indicates time point 24 h.

© 2020 The Authors

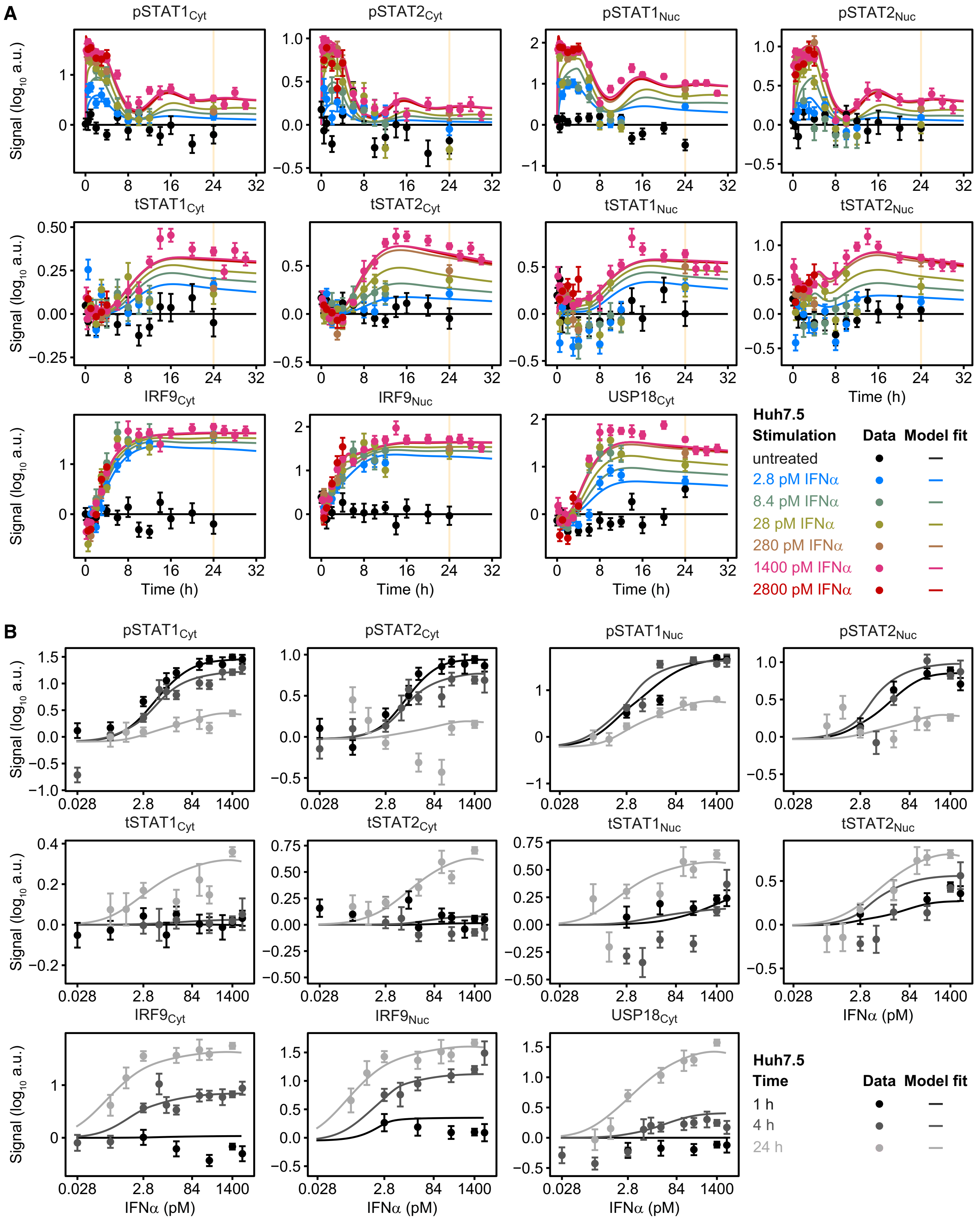

Figure EV1.

 © 2020 The Authors

Figure EV2. Quantitative data with different prestimulation doses for model calibration.

A Model calibration with IFNα-induced expression of feedback transcripts *STAT1*, *STAT2*, *IRF9*, *IRF2*, *USP18*, *SOCS1*, and *SOCS3*. Growth factor-depleted Huh7.5 cells were prestimulated with 2.8, 28, and 1,400 pM IFNα (yellow background). After 24 h, cells were stimulated with 1,400 pM IFNα or were left untreated (white background). IFNα-induced expression of target genes was measured by qRT-PCR. RNA levels were normalized to the geometric mean of reference genes GAPDH, HPRT, and TBP and were displayed as fold change, visualized by filled circles with errors representing 1σ confidence intervals estimated from biological replicates (N = 3 to N = 14) using a combined scaling and error model. Model trajectories are represented by lines.

B Growth factor-depleted Huh7.5 were prestimulated with a range of 0.28–1,400 pM IFNα for 24 h and stimulated with 1,400 or 2,800 pM IFNα. IFNα-induced phosphorylation of nuclear STAT1 and STAT2 and abundance of feedback proteins were analyzed by quantitative immunoblotting. Data were normalized to reference proteins Calnexin or HDAC1 and represented by filled circles with errors representing 1σ confidence intervals estimated from biological replicates (N = 1 to N = 19) using a combined scaling and error model. Model trajectories are represented by lines.

© 2020 The Authors

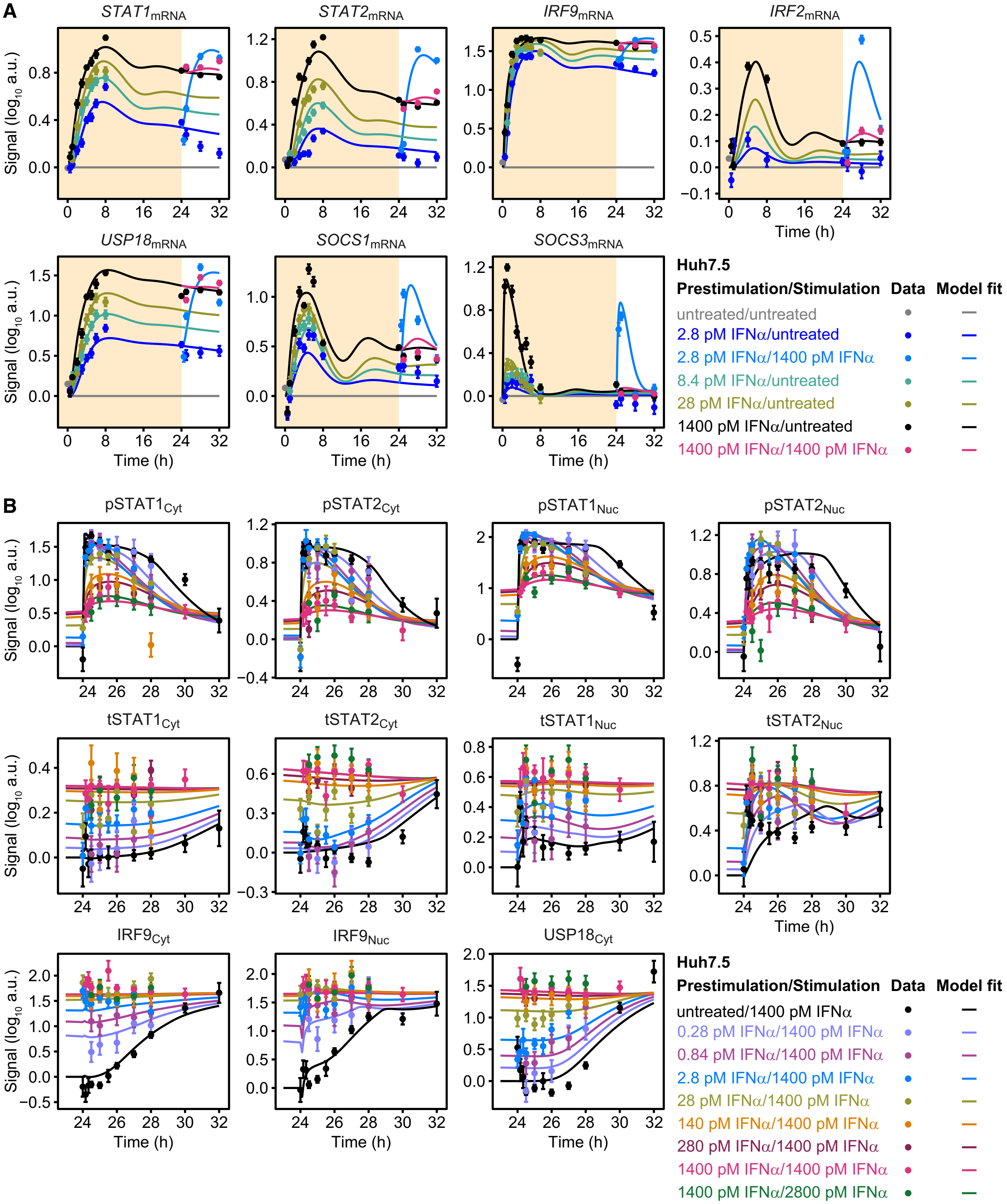

Figure EV2.

© 2020 The Authors

Figure EV3. Identification of GAS- and ISRE-driven interferon target genes and model validation using SOCS3 protein dynamics.

A Electrophoretic mobility shift assays were performed using nuclear lysates obtained from untreated Huh7.5 cells or Huh7.5 cells that were prestimulated for 24 h with 280 pM IFNα and then stimulated with 1,400 pM IFNα. Time points after prestimulation with IFNα are indicated. Nuclear lysates were incubated with radioactively labeled oligonucleotides probes harboring the GAS-binding region of the human IRF1 promoter. A representative image of an EMSA gel of $N = 3$ replicates is shown (left panel). For the supershift experiments, the mixture of DNA with nuclear lysates from untreated Huh7.5 cells that were stimulated with 1,400 pM IFNα for 1 h was incubated with antibodies against either STAT1, STAT2, or IRF9 (right panel). Samples were resolved on a native polyacrylamide gel, and radioactivity was visualized. Putative pSTAT1:pSTAT1 complexes are indicated.

B Co-immunoprecipitation experiments were performed using total cell lysates obtained from untreated Huh7.5 cells or Huh7.5 cells that were prestimulated for 24 h with 280 pM and then stimulated with 1,400 pM IFNα. Time points after prestimulation are indicated. Lysates were incubated with antibodies directed against STAT2 and subjected to immunoprecipitation. Phosphorylation of STAT1 was detected by quantitative immunoblotting utilizing an antibody that recognizes STAT1 phosphorylated on tyrosine residue 701. An asterisk indicates pSTAT1β. Membranes were re-probed with antibodies recognizing STAT2. Molecular weights are indicated on the left. Immunoblot detection was performed with chemiluminescence employing a CCD camera-based device (ImageQuant). A representative immunoblot of $N = 3$ replicates is shown.

C Co-immunoprecipitation experiments were performed using total cell lysates obtained from untreated Huh7.5 cells or Huh7.5 cells that were prestimulated for 24 h with 280 pM and then stimulated with 1,400 pM IFNα. Time points after prestimulation are indicated. Lysates were incubated with antibodies directed against IRF9 and subjected to immunoprecipitation. Phosphorylation of STAT1 was detected by quantitative immunoblotting utilizing an antibody that recognizes STAT1 phosphorylated on tyrosine residue 701. An asterisk indicates pSTAT1β. Membranes were re-probed with antibodies recognizing IRF9. Molecular weights are indicated on the left. Immunoblot detection was performed with chemiluminescence employing a CCD camera-based device (ImageQuant). A representative immunoblot of $N = 3$ replicates is shown.

D Model prediction of IFNα-induced dynamics of occupied GAS-bindings sites (OccGASbs) in Huh7.5 cells without prestimulation and in cells prestimulated for 24 h with 2.8 and 28 pM IFNα that were subsequently stimulated with 1,400 pM IFNα. Model predictions were performed using the prediction profile likelihood method. Lines with shading represent model predictions with 1σ confidence intervals.

E Model prediction and experimental validation of IFNα-induced dynamics of SOCS3 protein in Huh7.5 cells without prestimulation and in cells prestimulated for 24 h with 2.8 and 28 pM IFNα that were subsequently stimulated with 1,400 pM IFNα. Model predictions were performed using the prediction profile likelihood method. Lines with shading represent model predictions with 1σ confidence intervals. SOCS3 protein data were used for model validation but not for model calibration. Data are represented by filled circles with errors representing 1σ confidence intervals estimated from biological replicates ($N = 3$) using a combined scaling and error model.

© 2020 The Authors

© 2020 The Authors

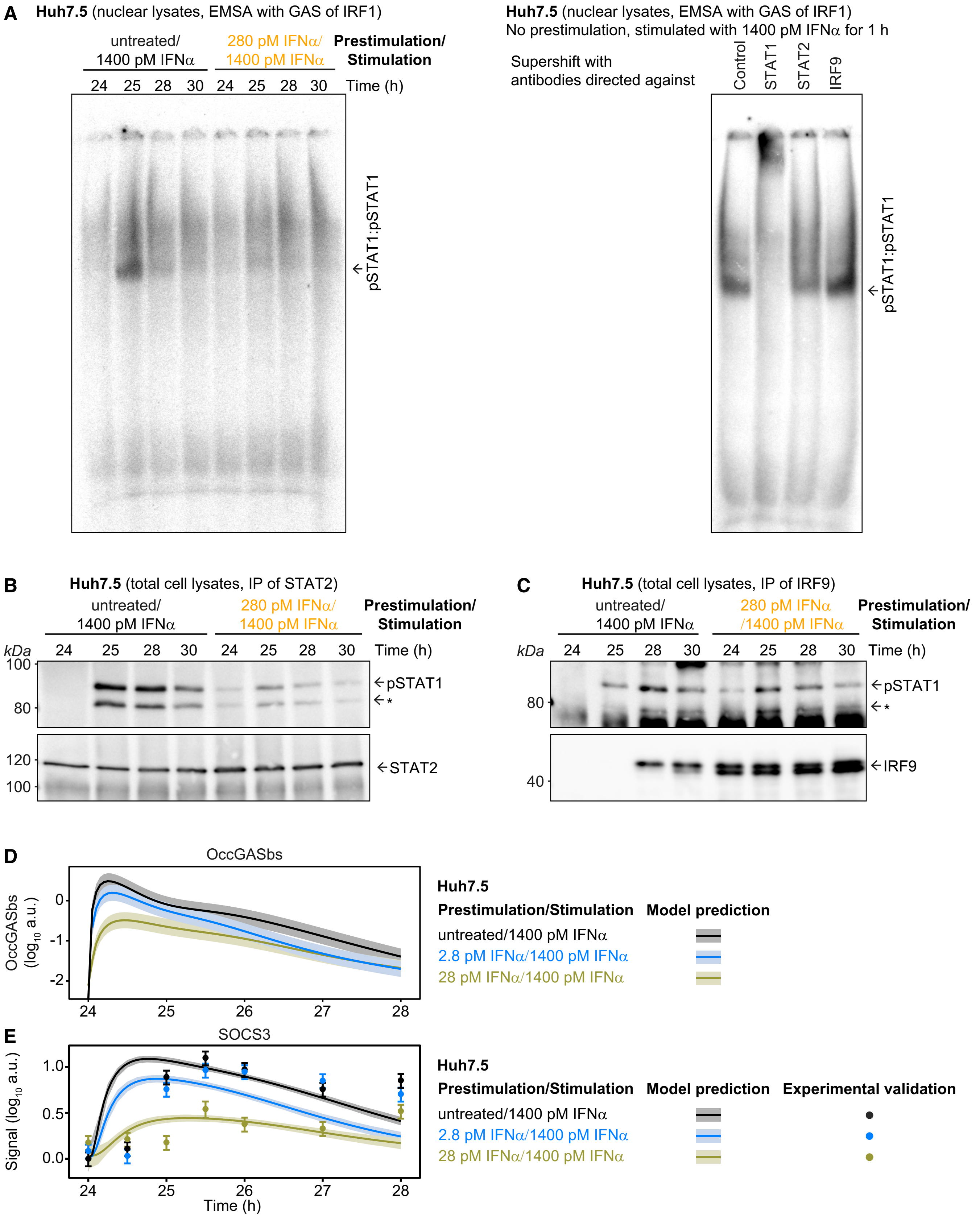

Figure EV3.

© 2020 The Authors

Figure EV4. Equipotent interferon concentrations and model calibration with Roferon-induced signal transduction in Huh7.5 cells.

A Huh7/LucUbiNeo cells harboring HCV replicon were treated with serial dilutions of IFNα or Roferon and luciferase units were determined. Data were approximated by sigmoidal regressions, and droplines indicate IC_{50} values.

B IC_{50} values as determined in (A) from two independent experiments for IFNα and Roferon are displayed with standard deviations. IC_{50} values were used to determine equipotent doses of IFNα and Roferon.

C Huh7.5 were stimulated with the indicated concentrations of Roferon. Cytoplasmic protein lysates were collected 1 h after the stimulation and phosphorylation of STAT1 and STAT2 was detected by immunoblotting. Dots represent scaled data with errors representing 1σ confidence intervals estimated from biological replicates (N = 3 to N = 4) using a combined scaling and error model. Model fit is shown with a solid line and signals corresponding to a low dose (1.2 pM Roferon) and a high dose (608 pM Roferon) are displayed with dashed lines.

D Dose-dependent sensitization of signal transduction induced by the therapeutic interferon Roferon. Growth factor-depleted Huh7.5 were prestimulated with 0, with 1.2 or 608 pM Roferon and stimulated with 608 pM Roferon 24 h later. Concentrations of Roferon correspond to equipotent concentrations of IFNα. Cytoplasmic and nuclear lysates were subjected to quantitative immunoblotting and Roferon-induced phosphorylation of STAT1 and STAT2 and induction of feedback proteins was detected by chemiluminescence utilizing a CCD camera-based device (ImageQuant). Dots represent scaled data with errors representing 1σ confidence intervals estimated from biological replicates (N = 3 to N = 8) using a combined scaling and error model. Model trajectories are represented by lines.

© 2020 The Authors

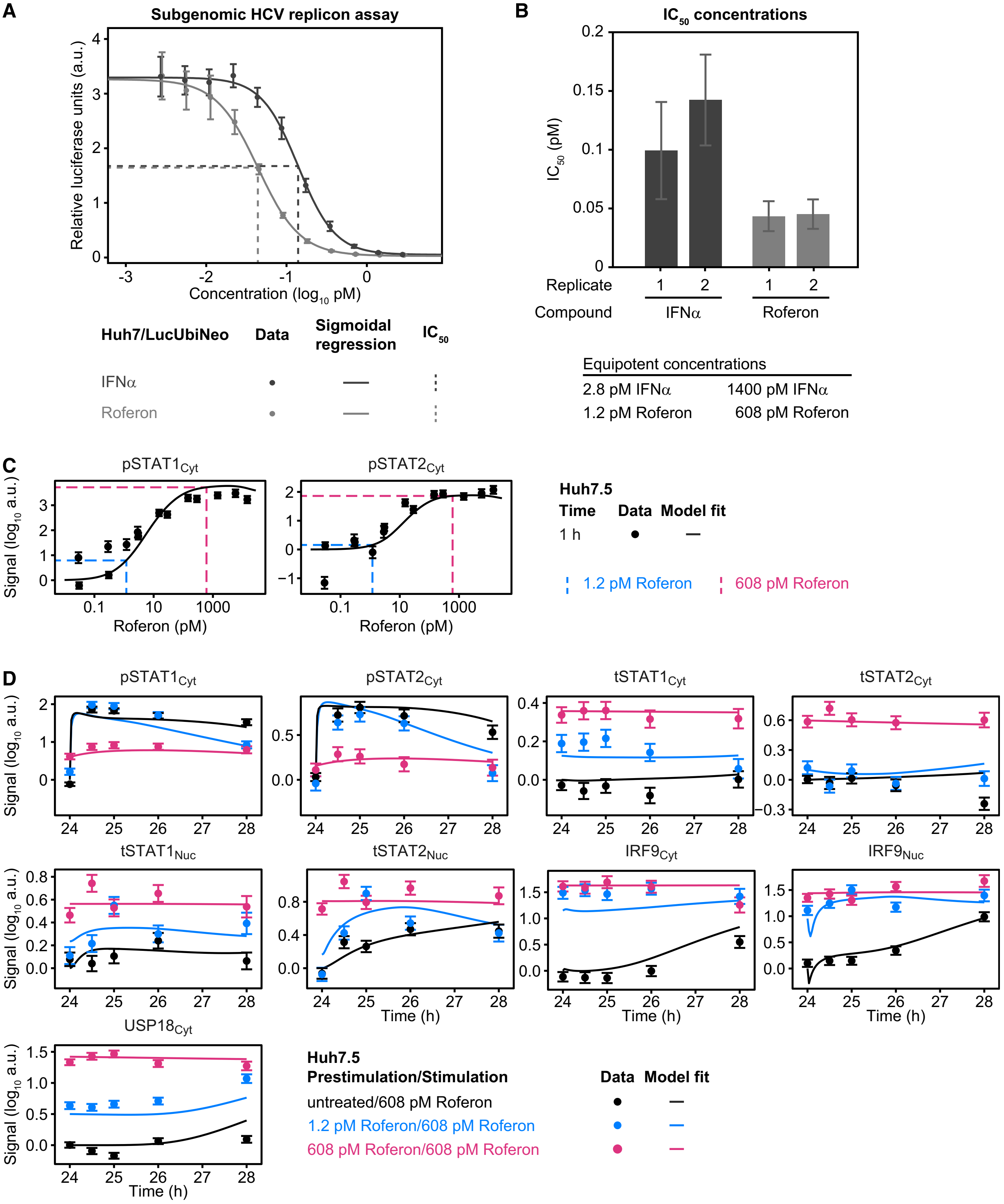

Figure EV4.

© 2020 The Authors

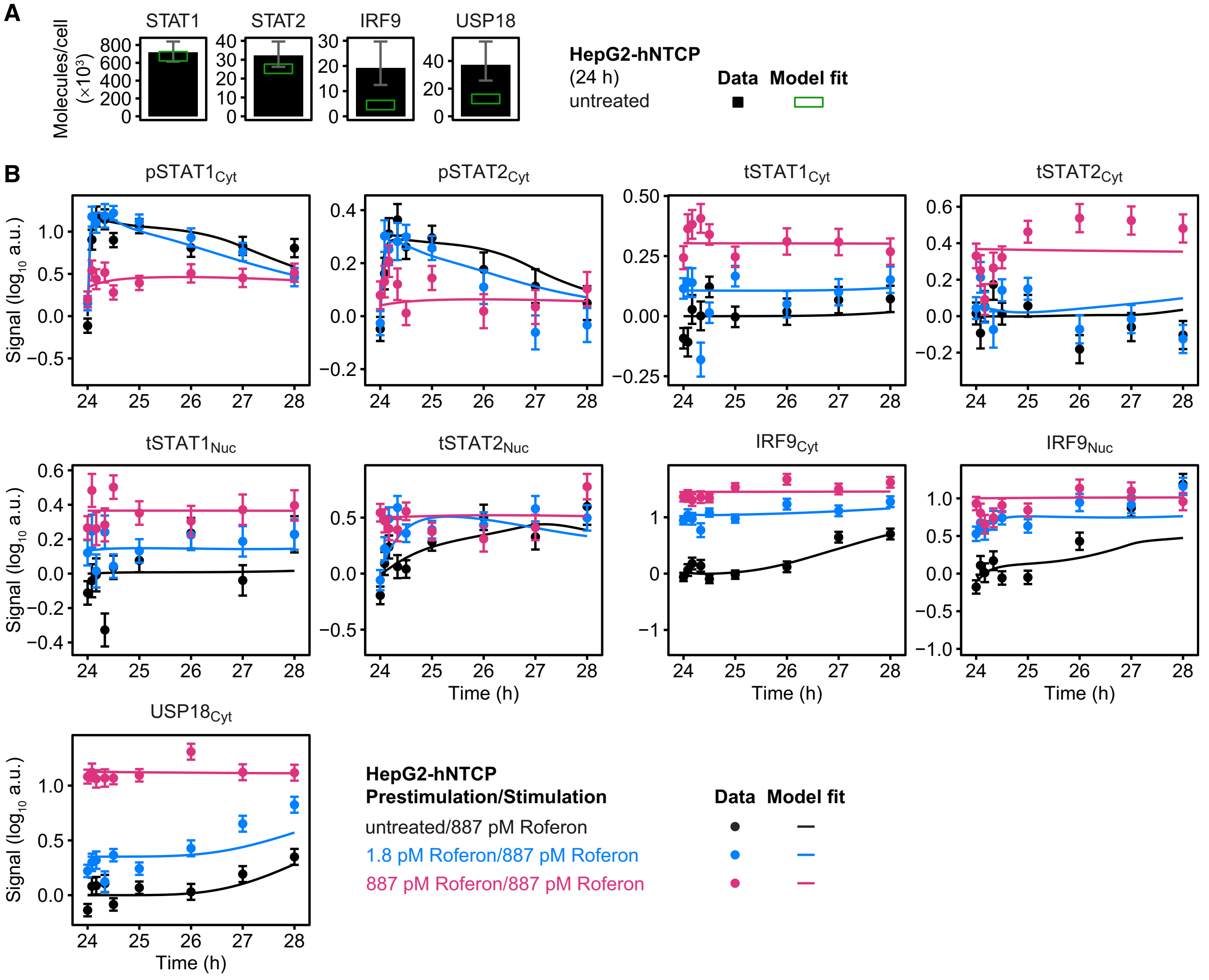

Figure EV5. Model calibration with amount of molecules per cell and Roferon-induced signal transduction in HepG2-hNTCP cells.

A Model calibration with the amount of molecules per cell of STAT1, STAT2, IRF9, and USP18 in untreated HepG2-hNTCP cells. Calibrator proteins were spiked into total protein lysates and subjected to quantitative immunoblotting. Immunoblot detection was performed by chemiluminescence using a CCD camera-based device. Averaged values (N = 4) are displayed with standard deviations. Green squares indicate amounts estimated by the mathematical model.

B Dose-dependent sensitization of signal transduction induced by Roferon in HepG2-hNTCP. Growth factor-depleted HepG2-hNTCP were prestimulated with 0, 1.8 or 887 pM Roferon and stimulated with 887 pM Roferon 24 h later. Cytoplasmic and nuclear lysates were subjected to quantitative immunoblotting and Roferon-induced phosphorylation of STAT1 and STAT2 induction of feedback proteins was detected by chemiluminescence utilizing a CCD camera-based device (ImageQuant). Dots represent scaled data with errors representing 1σ confidence intervals estimated from biological replicates (N = 3) using a combined scaling and error model. Model trajectories are represented by lines.

© 2020 The Authors
